# A functional cell model using basophil activation test to study molecular mechanisms and biomarkers of response to omalizumab treatment in patients with asthma

**DOI:** 10.3389/fimmu.2026.1744735

**Published:** 2026-02-27

**Authors:** Boris Gole, Larisa Goričan, Gregor Jezernik, Mario Gorenjak, Anja Bizjak, Vojko Berce, Maja Skerbinjek Kavalar, Michael Kabesch, Erik Melén, Korneliusz Golebski, Cornelius M. van Drunen, Anke Hilse Maitland-van der Zee, Susanne Reinartz, Susanne J. H. Vijverberg, Uroš Potočnik

**Affiliations:** 1 Center for Human Molecular Genetics and Pharmacogenomics, Faculty of Medicine, University of Maribor, Maribor, Slovenia; 2 Department of Pediatrics, University Medical Center Maribor, Maribor, Slovenia; 3 Ambulanta Čebelica, Maribor, Slovenia; 4 Department of Pediatric Pneumology and Allergy, University Children’s Hospital Regensburg (KUNO), Regensburg, Germany; 5 Department of Clinical Sciences and Education Södersjukhuset, Karolinska Institutet, Stockholm, Sweden; 6 Department of Pulmonary Medicine, Amsterdam University Medical Center, University of Amsterdam, Amsterdam, Netherlands; 7 Department of Otorhinolaryngology, Amsterdam University Medical Center, University of Amsterdam, Amsterdam, Netherlands; 8 Department of Pediatric Pulmonology, Emma Children’s Hospital, Amsterdam University Medical Center, University of Amsterdam, Amsterdam, Netherlands; 9 Department of Otorhinolaryngology, Tergooi Hospitals, Hilversum, Netherlands; 10 Laboratory for Biochemistry, Molecular Biology and Genomics, Faculty for Chemistry and Chemical Engineering, University of Maribor, Maribor, Slovenia; 11 Department for Science and Research, University Medical Center Maribor, Maribor, Slovenia

**Keywords:** basophil activation, biomarker, differential response to omalizumab, *in vitro* cell model, pediatric asthma, precision medicine, transcriptomics

## Abstract

**Introduction:**

Patients with severe asthma may benefit from targeted biological therapies. However, personalized therapy in children and adolescents with asthma, based on individual susceptibility to molecular mechanisms addressed by different biologicals is underdeveloped. Here, we established a functional *in vitro* model, to study the differential responses of asthma patients to omalizumab (an IgE targeting biological) therapy.

**Methods:**

White blood cells isolated from asthmatic children and adolescents were pre-treated with omalizumab. Next, basophil activation and degradation were used to assess the *in vitro* patient’s response to omalizumab after exposure to patient-specific allergens. In parallel, basophils-specific whole RNA sequencing was used to screen for differentially expressed genes associated with an *in vitro* response to omalizumab. The results of the screen were first confirmed in an independent cohort of patients, and finally compared to the clinically relevant data.

**Results:**

The *in vitro* basophil activation + degradation test may be used to study the differential response to omalizumab in patients. The differentially expressed genes in the basophils of the better vs. the poor/non-responders are associated primarily with defense against viruses. The low *RSAD2* expression correlates with poor response to omalizumab *in vitro*.

**Conclusions:**

We describe an *in vitro* test to study the differential response to omalizumab in patients. *RSAD2* may be a biomarker for response to omalizumab in asthma.

## Introduction

Asthma, a chronic inflammatory disease of the airways of the lungs, remains a major health challenge worldwide, particularly in the pediatric population (1, 2). 5-10% of children and adolescents with asthma show a poor response to standard therapies, including inhaled corticosteroids (1, 3). These patients with severe asthma represent a significant clinical challenge, highlighting the urgent need for alternative therapeutic strategies.

A promising avenue for the treatment of severe asthma has emerged with biological therapies. The first biological therapy for corticosteroid resistant asthma was omalizumab. This humanized monoclonal antibody targeting immunoglobulin E (IgE) has revolutionized the treatment paradigm for severe allergic asthma (4, 5). By binding to the circulating IgE, omalizumab attenuates the IgE-mediated inflammatory responses, thereby reducing the severity of the asthma symptoms (6). However, despite its therapeutic promise, some 20-25% of patients fail to respond to this treatment, highlighting the heterogeneous nature of asthma pathogenesis and the limitations of a one-size-fits-all approach (1, 7). Identification of suitable biomarkers to predict omalizumab (non-) response would allow a more personalized approach, potentially improving the clinical outcomes of pediatric patients with asthma and reduce number of cases with unnecessary treatment (1, 7). To this end, basophils have emerged as a promising cellular target (8). These immune cells, activated in the context of allergic reactions, have been shown to undergo significant phenotypic and functional changes (decreased levels of the FcϵRIα receptor, reduced production of Th2 cytokines) in patients responding to omalizumab therapy (9, 10).

There is an unmet need to develop *in vitro* human cell models that can predict clinical response to biologics and support the search for candidate predictive biomarkers (11–13). In the presented work we have developed an *in vitro* primary cell model using basophils to study the cellular and molecular response to omalizumab in young patients with allergic asthma. The model combines an innovative use of the Basophil Activation Test (BAT) and high-throughput cell type-specific transcriptomic analysis to identify the potential biomarkers of (non-) responsiveness to omalizumab.

## Materials and methods

### Donors and sample processing

The study was approved by the National Medical Ethics Committee of the Republic of Slovenia (approval 0120-187/2019/10) and the Swedish Ethical Review Authority (approval 2019-05260). Written informed consent was obtained from all the donors (their legal guardians) who participated in the study.

Three separate cohorts of patients were recruited. For the discovery and validation cohorts we recruited Slovenian patients with allergic asthma, GINA (Global Initiative for Asthma) steps 1-4, not (yet) eligible for the biological therapy (**Supplementary Table S1**). The age range of the patients was 6–20 years, and included children above 6 years of age (the minimum age to be eligible for omalizumab treatment) (14) and adolescents/young adults (within the age range of 10–24 years) (15). A confirmed allergy to either grass pollen or house dust mites was an additional inclusion criterion. Patients were excluded with current asthma exacerbation or symptoms of acute respiratory infection. The clinical cohort was comprised of Swedish patients with allergic asthma, multiple confirmed allergies, aged 9–16 years, GINA steps 2-5, who were selected for omalizumab therapy (**Supplementary Table S2**).

From the discovery and validation cohort patients 12 mL of venous blood was collected into K2EDTA tubes (BD Vacutainer, BD, New Jersey, USA). The blood plasma was separated by centrifugation at 300×g, 20 min, room temperature (RT); next, the white blood cells (WBC) were isolated by gradient centrifugation (1.200×g, 10 min, RT) using a density medium (1.094 g/mL, mixed from High Density Spin Medium and Density Diluent Medium, both pluriSelect, Leipzig, Germany) and SepMate50 tubes (Stem Cell Technologies, Vancouver, Canada).

From the clinical cohort patients, 2.5 mL of venous blood was collected into the PAXgene RNA tubes (BD Vacutainer), and stored at -20 °C within 2h of collection, and then at -80 °C within 24h after freezing at -20 °C. The samples were stored at -80 °C until RNA isolation.

### The cell model and pre-treatment

**Figure 1:**
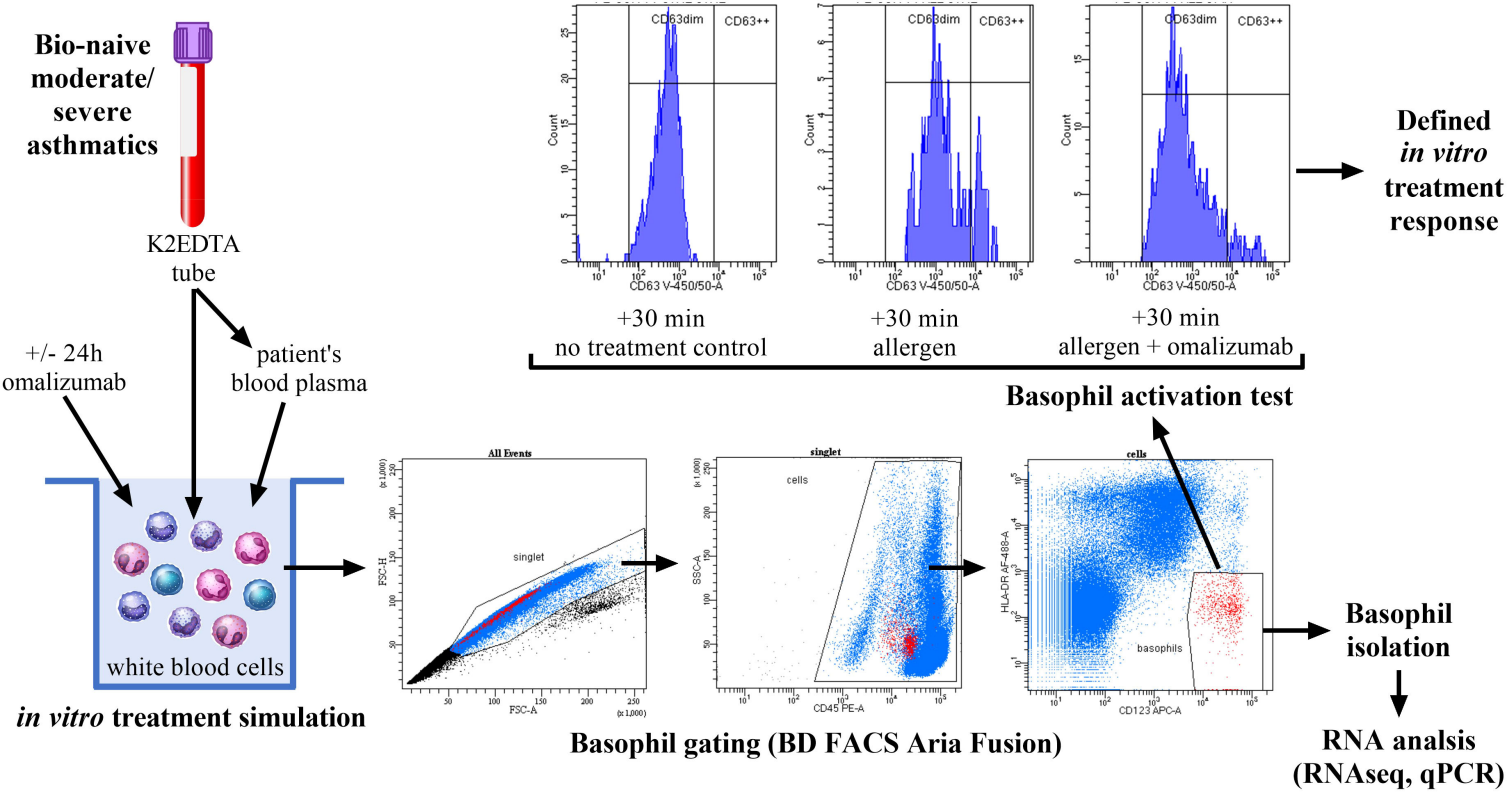
The experimental setup. Venous blood was drawn from children/juveniles with moderate to severe asthma, naive to biologic therapy. The total White Blood Cells (WBC) were isolated using density gradient centrifugation, and then cultured *in vitro* for 24h with/without omalizumab in media containing the patients’ blood plasma. Next, the samples were split into two parts. The first part was used for basophil isolation (CD45^+^CD123^+^HLA-DR^neg^) by FACS sorting and subsequent cell-type (basophil) specific RNA analysis (RNAseq, qPCR). The second part was used for the Basophil Activation Test (BAT). These cells were exposed to a donor-specific allergen for 30 minutes and stained for basophils expressing CD63 on their surface (CD45^+^CD123^+^HLA-DR^neg^CD63^++^). The BAT results and the % of basophils were used to assess the *in vitro* response to omalizumab.

The freshly isolated WBC from the discovery and validation cohorts were seeded in RPMI-1640 media (Gibco, ThermoFisher Scientific, Waltham, MA, USA) supplemented with 10% of the respective donor’s own blood plasma and 1% penicillin/streptomycin (Sigma-Aldrich, St. Louis, MO, USA) at a density of 2.5×10^6^ cells/mL. One half of the cells were treated with 3.32 µg/mL omalizumab (Xolair, Novartis, Basel, Switzerland) for 24 hours at standard cell culture conditions - 37 °C, 5%CO_2_, >95% relative humidity (**Figure 1**). The specific concentration of omalizumab used was calculated from the manufacturer’s recommended dose for subcutaneous administration to patients (14).

### Isolation of the basophils

The basophils were isolated using fluorescence activated cell sorting (FACS Aria Fusion, BD Biosciences, New Jersey, USA). First, the single cells (singlets) were gated using the FSC-A/FSC-H gating. Next, the CD45^+^ leukocytes (cells) were defined using the CD45/SSC plot, and, finally, a CD123/HLA-DR plot was used to gate for the CD123^+^HLA-DR^neg^ population, corresponding to the basophils. The representative plots are shown in **Figure 1**. The following conjugated primary antibodies were used: Mouse IgG anti-human CD45-PE (#1P-160-T100), Mouse IgG anti-human CD123-APC (#1A-700-T100) and Mouse IgG anti-human HLA-DR-FITC (#1F-474-T100, all EXBIO, Prague, Czechia). Antibody concentrations were used recommended by the manufacturer. On a subset of samples, the sorting efficacy was confirmed via flow cytometry of the sorted samples (**Supplementary Figure S1A**).

### The basophil activation test and assessment of the *in vitro* response to omalizumab

Following the 24-hour incubation, parts of the omalizumab pre-treated and control (no pre-treatment) WBC were used to perform BAT using a patient-specific allergen- either 1.0 µg house dust mite allergen (#ALYOST46) or a 10.0 µg grass pollen allergen mix composed of 5 different types of grass species- *i.e.*, 2.0 µg of each allergen (#ALYOS106, both Stallergenes Greer, Baar, Switzerland) for 30 minutes. The concentrations correspond to the ones used for the clinical testing with BAT at the local medical center (University Medical Center Maribor, Slovenia). 180 IU/mg heparin and 20 µM CaCl_2_ (both Sigma-Aldrich) were added to the media together with allergens, to mitigate cell aggregation and enable basophil activation, respectively.

At the same time, the cells were also stained for the basophils-specific markers (see Isolation of the basophils) and CD63 (Mouse anti-Human CD63-V450, #561984, BD Biosciences), to determine the % of the total and of the CD63-expressing (CD45^+^CD123^+^HLA-DR^neg^CD63^++^) basophils on the flow cytometer (**Figure 1**). The BAT results were calculated as the % of the basophils expressing CD63 on their surface (CD63^++^ basophils) in the total basophil population.

A corrected activity readout was used to assess the *in vitro* patients’ response to omalizumab. For this, the proportion of CD63-expressing basophils was normalized to the total WBC as a % of the CD63^++^ basophils among the CD45^+^ leukocytes. Next, the basophil loss (missing basophils) was quantified as the decrease in the % of total basophils in the allergen challenged samples relative to the corresponding samples without the allergen challenge. These calculations were performed separately for omalizumab treated and untreated conditions. Then, the proportion of missing basophils was added to both the total and the CD63^++^ basophil count in the allergen challenged samples. The final readout was defined as the proportion of activated basophils (CD63^++^ basophils + missing basophils) in (total basophils + missing basophils). All the fold changes of basophil activity (allergen vs. no treatment, allergen + omalizumab vs. allergen) were calculated from these corrected activity readouts.

### RNA isolation, RNA sequencing, and quantitative real-time PCR

The RNA was isolated from the sorted basophils (the *in vitro* testing) using the RNeasy Plus Mini Kit (Qiagen, Hilden, Germany) and from PAX RNA tubes (the clinical cohort) using the PAXgene Blood miRNA Kit (PreAnalytiX, Hombrechtikon, Switzerland), following the manufacturers’ recommendations. The RNA isolated from the PAX RNA tubes was depleted using the GLOBINclear Human Globin mRNA Removal Kit (ThermoFisher Scientific, Waltham, MA, USA). The concentration was determined using the Synergy2 microplate reader (Biotek, Winooski, VT, USA), and the RNA`s integrity was assessed using the Bioanalyzer 2100 (Agilent Technologies, Waldbronn, Germany) or by gel electrophoresis.

For the RNA sequencing (RNAseq), cDNA libraries were prepared using the Stranded mRNA Prep Ligation Kit (Illumina, San Diego, CA, USA) according to the manufacturer’s instructions. Paired-end RNA sequencing (2×74 bp) was performed on a NextSeq 550 system (Illumina) using a NextSeq 500/550 Mid Output Kit v2.5 (Illumina). The raw FASTQ data were processed as previously described (16). An RNAseq data analysis was performed as described previously (17) in the R programming environment (R Core Team, Vienna, Austria). Importantly, mean–variance modeling at the observation level transformation (VOOM) (18), followed by linear models and empirical bayes in the limma R package (19), were applied to obtain the differentially expressed genes between the groups. The differential gene expression was calculated as a log_2_-fold change in expression between the *in vitro* better and poor/non-responders to omalizumab treatment.

For the qRT-PCR, the cDNA was generated using the High-Capacity cDNA Reverse Transcription Kit (Applied Biosystems, Thermo Fisher Scientific, Waltham, MA, USA). The qRT-PCR was performed with LightCycler480 SYBR Green I Master (Roche, Mannheim, Germany) on a QuantStudio 12 K Flex Real-Time PCR System (Applied Biosystems). The forward and reverse primers used to detect gene-specific mRNA were designed with the Primer3 software, version 4.1.0, and produced by Sigma-Aldrich. The primer sequences are listed in the **Supplementary Table S3**. The gene expression was calculated using the 2^−ΔΔCt^ method. Ct values above 40 were considered negative. The geometric mean value of the beta-2-microglobulin (*B2M*), glyceraldehyde 3-phosphate dehydrogenase (*GAPDH*) and 18S ribosomal N5 RNA (*RNA18SN5*) was used as the internal control.

### Construction of the interactome, gene ontology analysis, comparison of the signaling pathways and basophil-specific expression

The interactome of the proteins coded by the differentially expressed genes found with the RNAseq analysis was built with the CytoScape platform (version 3.10.3) (20), based on the protein–protein interactions downloaded from the BioGrid database (build version 5.0.250; https://thebiogrid.org/). The non-protein coding genes were inserted into the interactome manually.

A gene ontology (GO) analysis was performed using the ShinyGO 0.85 gene-set enrichment tool (21), with the following parameters and selected options: comparison of the selected gene set against a background of all the genes detected by the above RNAseq, GO-terms from the GO Biological Process database with at least 3, and no more than 500, associated genes considered, enrichment calculated based on hypergeometric distribution followed by the false discovery rate (FDR) correction. Genes of interest for downstream testing were selected based on the above GO analysis.

### Statistical analysis

All the data are presented as the mean ± standard error. Statistical analyses of the qRT-PCR and the clinical data were performed using the GraphPad Prism software (GraphPad Software Inc., San Diego, CA, USA). A D’Agostino-Pearson omnibus normality test showed that most of the data sets had abnormal distributions. Hence, to assess the statistical significance of the differences between the two groups, a Wilcoxon matched pairs test, or the nonparametric Mann-Whitney test were used in cases of paired or unpaired data, respectively. The nonparametric Spearman r was calculated to assess the correlations between the datasets. A receiver operating characteristic (ROC) analysis was calculated using the Wilson/Brown method and a 95% confidence interval. The significance limit was set at p<0.05 for all the tests. A Benjamini-Hochberg step-up procedure (FDR) was used to adjust the p-values for multiple testing where applicable. Where mentioned specifically, the data outliers were determined using Tukey’s IQR rule and excluded from the analysis.

## Results

### Establishing the *in vitro* model

To establish an *in vitro* model for testing the patients’ response to omalizumab, we used BAT. In clinics, BAT is used typically to confirm a patient’s allergic response to a specific allergen. The patient’s WBC are exposed to the tested allergens, and a positive result is defined as >5% of basophils expressing CD63 on their surface (CD63^++^ basophils) (22). For our model, we used allergens (grass pollen mix, house dust mites) to which the patients were confirmed to be allergic, *i.e.*, patient-specific allergens that guaranteed a positive BAT result, whereby the intensity of the basophil activation is dependent on the allergen’s concentration (**Supplementary Figure S1B**). It turns out that the 1× concentration (*i.e.*, the concentration optimized for clinical testing at the local medical center, see Materials and Methods section) is indeed the one that gives the maximum activation. On the other hand, when an allergen to which the patient is not allergic is used, the percentage of CD63^++^ basophils remained similar as in no treatment control, even when the concentration of the allergen was doubled (**Supplementary Figure S1B**).

Next, we made sure that the WBC samples from allergic asthmatics contained cells (basophils) with free FcϵRI not occupied by IgE (**Supplementary Figure S1D**), confirming that omalizumab could have a measurable effect in our experimental setting. Namely, omalizumab blocks free IgE in a patient’s blood/serum, and prevents it from binding to the FcϵRI receptors on the surface of the basophils, thus preventing their activation when exposed to the corresponding allergen. It cannot, however, block IgE already bound to the FcϵRI (23). Indeed, in our model, pre-treatment of the WBC samples with omalizumab prior to the challenge with the patient-specific allergen resulted in a reduced % of CD63^++^ basophils upon the challenge, but had no major effect on the % of CD63^++^ basophils in the control (no allergen challenge) sample (**Supplementary Figure S1C**).

After confirming the feasibility of our *in vitro* setting, we used samples from 6 juvenile asthmatics (3 males, 3 females, mean age 14.2 years, GINA steps 1-3, **Supplementary Table S1**) with a confirmed allergy to either grass pollen or house dust mites to test it (the discovery cohort). In all 6 cases, exposure of the WBC to the patient-specific allergen increased the share of CD63^++^ basophils markedly (**Supplementary Figure S2A**). While analyzing the numbers of basophils though, we found that the allergen exposure not only increased the proportion of CD63^++^ basophils, but, in many cases, also decreased the total proportion of basophils in the WBC (**Supplementary Figure S2B**). Such a phenomenon was described previously, and may be attributed to strong activation and subsequent extensive degranulation, cell death or structural collapse, and, thus, changed scatter characteristics on the flow cytometer (24). We used a reverse gating strategy to test whether such a deleterious over-activation effect had occurred in our setting. We gated the total events (WBC) for the basophil-specific markers (CD45^+^CD123^+^HLA-DR^neg^) in two steps, and then used the FSC-A/FSC-H dot plot to discriminate between the intact cells (*i.e.*, cells with the expected scatter characteristics) and the cells with altered scatter characteristics (**Supplementary Figure S2C**). It turned out that, on average, 6.57 ± 2.23% of the CD45^+^CD123^+^HLA-DR^neg^ events (putative basophils) were present in the altered scatter characteristics gate under control (no treatment) conditions, which doubled to 14.73 ± 4.32% after exposure to the patient-specific allergen (**Supplementary Figure S2D**), while a handful of events was scattered outside the defined gates. The omalizumab pre-treated samples had, on average, a similar percentage of the putative basophils with altered scatter characteristics as those without omalizumab pre-treatment (7.10 ± 2.02% without allergen challenge, 14.10 ± 3.99% with allergen challenge). Looking at the individual donors, the allergen challenge increased the % of putative basophils with altered scatter characteristics in 5/6 samples (mean Fc 2.73 ± 0.60%), while the omalizumab pre-treatment counteracted this partially in 4/6 samples (mean Fc 1.08 ± 0.27%, **Supplementary Figure S2E**). As this result confirmed that some of the basophils in our experimental setting may indeed alter the scatter characteristics after the allergen challenge, we included the information on the percentage of “missing” basophils in our assessment of the omalizumab (non)-response.

**Figure 2:**
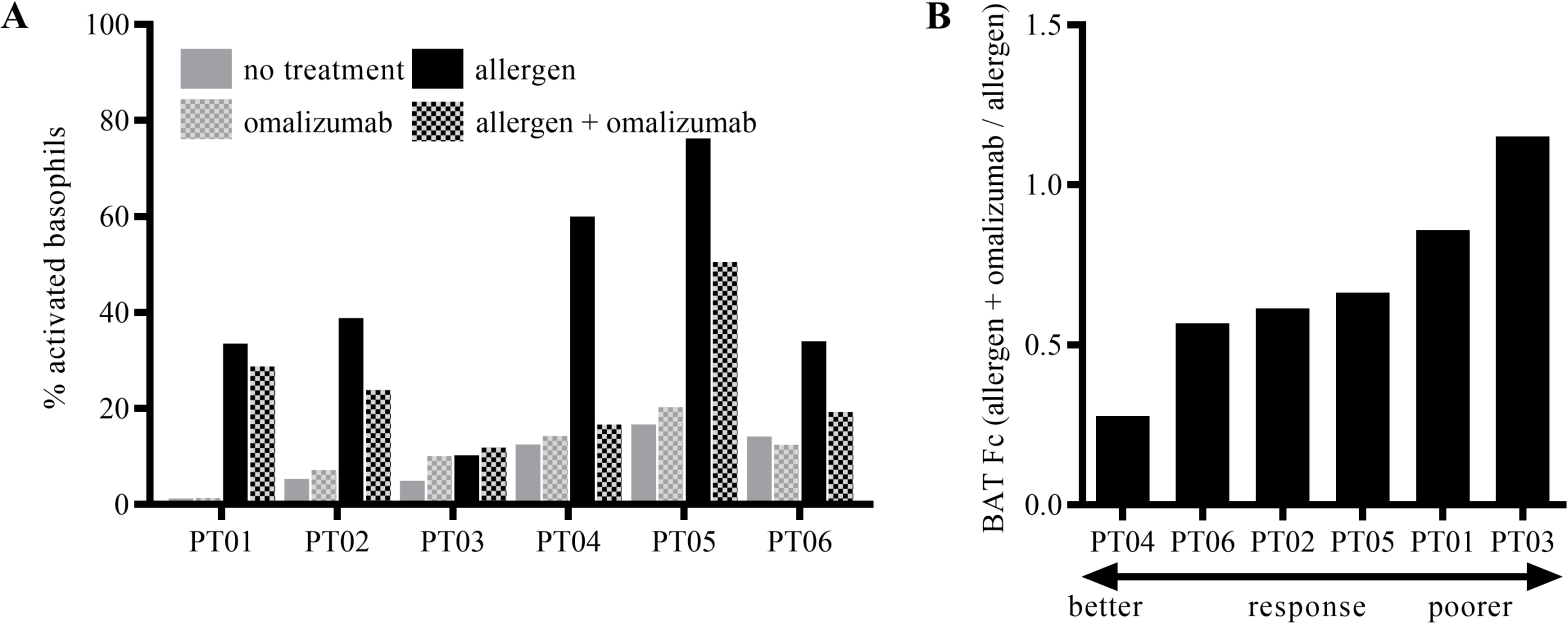
The basophil activation test model in the discovery cohort. **(A)** % of activated basophils in the control (no treatment) and omalizumab pretreated WBC samples after exposure to patient-specific allergens. **(B)** Fold changes (Fc) in the % of activated basophils in the WBC samples pretreated with omalizumab and exposed to patient-specific allergens vs. WBC samples exposed to patient-specific allergens but not pretreated with omalizumab. A lower BAT Fc = better response. The patients were divided into two equal cohorts of better and poor/non-responders.

Thus, the allergen exposure increased the % of activated (CD63^++^ + missing) basophils from ~1.3 – 16.7% to 10.3 – 76.3% (a 2.07 – 26.3-fold increase, mean increase 7.89 ± 3.75-fold; **Figure 2A**). The omalizumab pre-treatment had a varied effect, sometimes decreasing the share of the activated basophils after the allergen challenge, and sometimes not (Fc 0.28 - 1.15, mean Fc 0.69 ± 0.12, **Figure 2B**). This effect of omalizumab was independent of the patient’s age, sex, GINA step, ACT score, therapy, allergen used, or the fold-change in the activated basophils after the allergen challenge (**Supplementary Figure S3A-H**). These results thus confirm the concept that the variable response to omalizumab treatment can be measured in an *in vitro* experimental setting independent of the actual therapy in a patient (25). Based on the fold change in the percentage of activated basophils from allergen to allergen + omalizumab treated samples (BAT Fc), the 6 patients were ranked into two equal-sized groups (3 better and 3 poor/non-responders to omalizumab), in order to enable downstream comparative transcriptomic analysis.

### Transcriptome analysis of basophils from the samples with differential response to omalizumab

**Figure 3:**
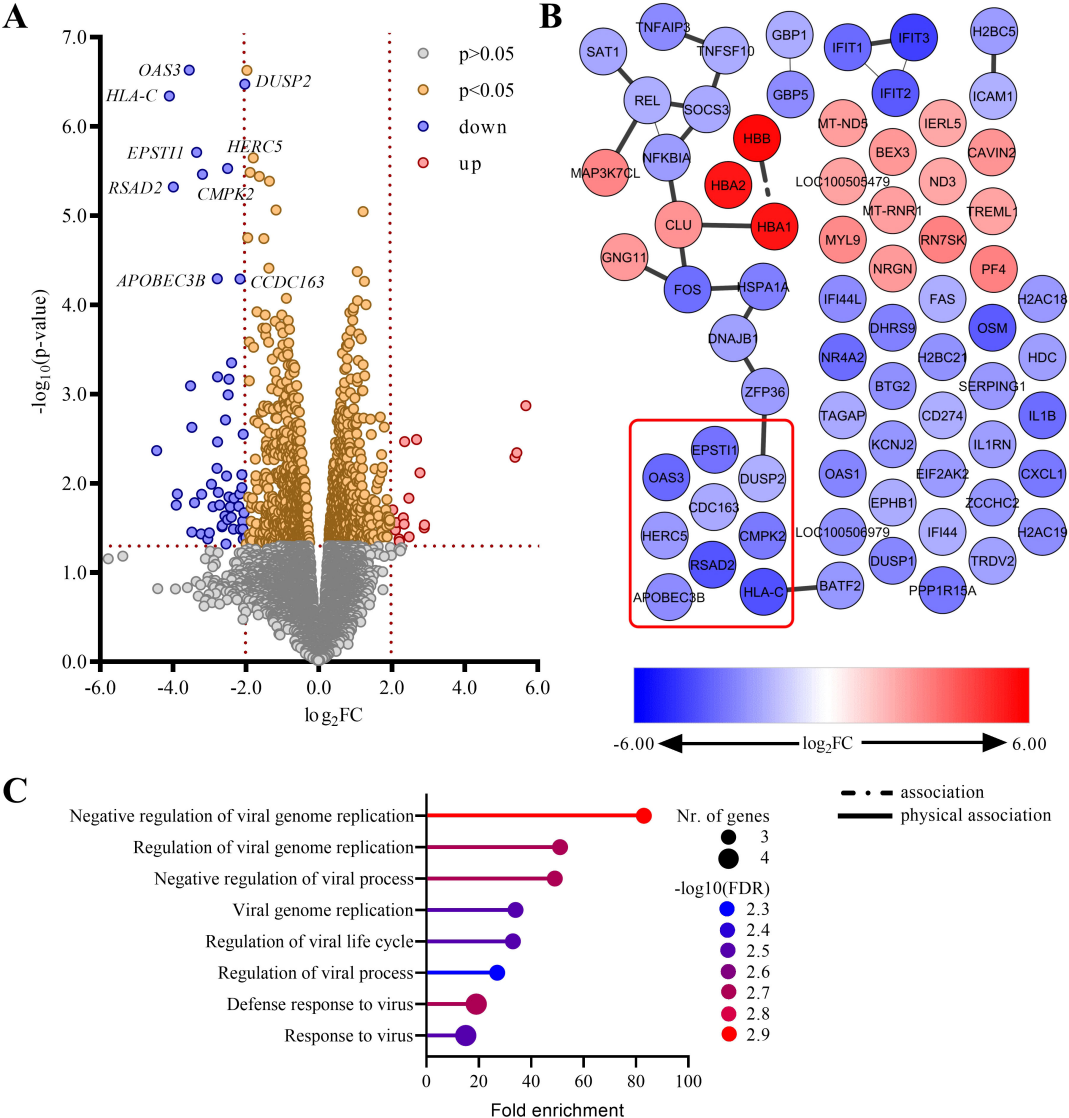
Transcriptomic analysis of the better and the poor/non-responders. **(A)** A volcano plot representing the RNAseq data. Log_2_FC>0.0 indicates increased and Log_2_FC<0.0 decreased expression in the poor/non-responder group compared to the better responder group. The significance levels were set at |log_2_FC|>2.0, p<0.05. The most prominent genes (with adjusted p<0.05) are highlighted. **(B)** The interactome of the 71 genes expressed differentially between the better and the poor/non-responder groups. The most prominent genes (with adjusted p<0.05) are highlighted by the red rectangle. **(C)** GO analysis of the 9 differentially expressed genes with an adjusted p<0.05 - lollipop diagram of the significantly enriched GO terms with FDR p<0.01.

In parallel to the allergen challenge, we also isolated the basophils from the same WBC samples via the FACS (mean purity 97.2 ± 1.7%, **Figure 1**; **Supplementary Figure S1A**), and used them for the cell type-specific whole transcriptome analysis (RNAseq). The expression profiles were compared of the basophils from the better and the poor/non-responder group of patients. 71 genes were expressed differentially (*i.e.*, |log2FC|>2.0, p<0.05; **Figure 3A**; **Supplementary Table S4**). 18 genes were upregulated, most prominently *HBB*, *HBA1* and *HBA2* (log_2_FC>5.0) and 53 genes were downregulated. Among the latter, for 9 genes- *APOBEC3B*, *CCDC163*, *CMPK2*, *DUSP2*, *EPSTI1*, *HERC5*, *HLA-C*, *OAS3* and *RSAD2* the p-value also remained significant after the adjustment for multiple testing (**Supplementary Table S4**). No upregulated genes remained significant after the adjustment. The interactome of the 71 genes revealed a single major network with 16 nodes representing individual genes, while the majority of the nodes (46 nodes, of which four represent non-protein coding genes) had no direct connections to each other (**Figure 3B**). Among the nodes with no direct connections in the interactome were also 7/9 genes with the adjusted p<0.05 (*APOBEC3B*, *CCDC163*, *CMPK2*, *EPSTI1*, *HERC5*, *OAS3* and *RSAD2*), while *HLA-C* was part of a small two-node network, and only *DUSP2* belonged to the single major network.

Next, we performed a Gene Ontology (GO) analysis of the 9 differentially expressed genes with adjusted p<0.05. The analysis revealed 96 over-represented GO terms (**Supplementary Table S5**), however, associated mostly with only one or two of the nine genes (69 terms and 19 terms, respectively). Only 9 GO terms were associated with more (3, 4) genes, of which 8 had an FDR p<0.01. All of these 8 GO terms are connected to a (negative) regulation of the viral life cycle/(defense) response to a virus (**Figure 3C**; **Supplementary Table S5**), and associated with the same four of the differentially expressed genes with the adjusted p<0.05- *APOBEC3B*, *HERC5*, *OAS3* and *RSAD2*, so these genes were identified as the most interesting targets for further study.

### *RSAD2* expression correlates negatively with the *in vitro* response to treatment with omalizumab

**Figure 4:**
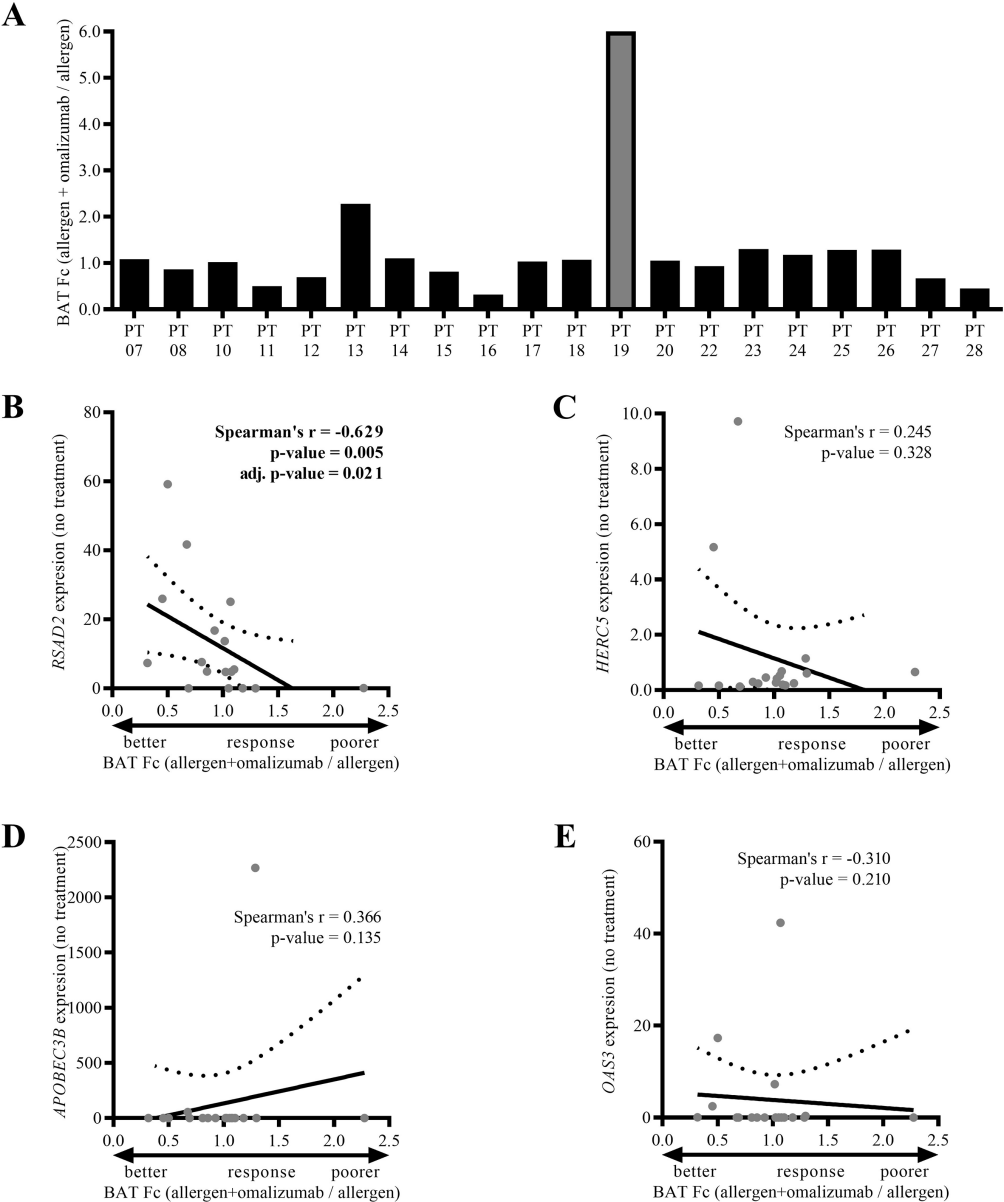
Validation of the selected target genes in the validation cohort. **(A)** Fold changes in the % of activated basophils in the WBC samples pretreated with omalizumab and exposed to patient-specific allergens vs. WBC samples exposed to patient-specific allergens but not pretreated with omalizumab (BAT Fc). **(B–E)** Spearman’s ranked correlations of the BAT Fc results (A lower BAT Fc = better response) and the **(B)***RSAD2*, **(C)***HERC5*, **(D)***APOBEC3B* and **(E)***OAS3* expression in the untreated basophils.

To test our *in vitro* model further and analyze the above most interesting target genes, an additional 22 juvenile asthmatics (16 males, 6 females, mean age 13.8 years, GINA steps 1-4, **Supplementary Table S1**) with confirmed allergy to either grass pollen or house dust mites were recruited as the validation cohort. The same as in the discovery cohort, the WBC were isolated, pretreated with omalizumab/no treatment, and then exposed to patient-specific allergens. Again, exposure of the WBC to the patient-specific allergens increased the proportion of the CD63^++^ basophils markedly, and in practically all cases decreased the total number of basophils (**Supplementary Figures S2A, B**, respectively). In the samples from patients PT09 and PT21 the allergen challenge did not increase the proportion of the CD63^++^ basophils above 5% (*i.e.*, no positive reaction), hence, the two samples were excluded from further analysis. As with the discovery cohort, pretreatment with omalizumab had a varied effect on the fold change in the % of activated basophils between omalizumab pre-treated + allergen challenged samples vs. allergen challenged samples with no omalizumab pre-treatment (Fc 0.32 – 6.00, mean Fc 1.25 ± 0.27, **Figure 4A**). The results were independent of the patient’s age at inclusion, sex, GINA step, ACT score, FeNO level, comorbidities (allergic conjunctivitis, atopic dermatitis), regular therapy, application of immunotherapy, allergen used in the test, or the fold-change in activated basophils after the allergen challenge (**Supplementary Figure S4C-K**). Of note, the BAT Fc result in patient 19 (PT 19) deviated markedly from all the others (**Figure 4A**). Since Tukey’s IQR rule identified this result as an outlier, we excluded this sample from the further analysis.

As before, in parallel to the allergen challenge and omalizumab response assessment, basophils were isolated from the same WBC samples, and used for the qPCR validation of the four most interesting target genes. As the separation of the patients/samples into the better and poor/non-responders is necessarily at least partially arbitrary, given the lack of standardized cut-offs for response to omalizumab *in vitro*, we focused primarily on correlation analyses, while using better/poor responder categorization only for the complementary analyses. We checked whether the expression of the four genes correlated with the response of the cells to the omalizumab pre-treatment after the allergen challenge (the BAT Fc results). Indeed, the expression of *RSAD2* in the basophils without omalizumab pretreatment correlated with the BAT Fc results significantly negatively, also after adjusting the p-value for multiple testing (Spearman r = -0.629, p = 0.005, adjusted p = 0.021, **Figure 4B**), which is consistent with the RNAseq results. No significant correlation between *HERC5*, *APOBEC3B* or *OAS3* expression and the BAT Fc results were found (**Figures 4C-E**). Additionally, based on a margin derived from the rank-based separation of the discovery cohort into two equal size groups (Fc ~0.6), we explored the group-wise differences in the validation cohort, which yielded only 3 better responders (PT 11, 16 and 28). In this small group, the mean baseline *RSAD2* expression in the basophils showed a trend toward higher expression compared to the poor/non-responder group (p=0.053, **Supplementary Figure S5A**). Further, the exploratory ROC analysis suggested a discriminatory capacity of the pre-treatment *RSAD2* expression in the basophils (AUC = 0.867, 95% CI 0.667 – 1.000), although the statistical significance was borderline (p=0.051, **Supplementary Figure S5B**). The detailed threshold-based performance metrics are provided in **Supplementary Table S6**.

### Whole blood *RSAD2* expression in a small clinical cohort of juvenile asthma patients starting omalizumab therapy

**Figure 5:**
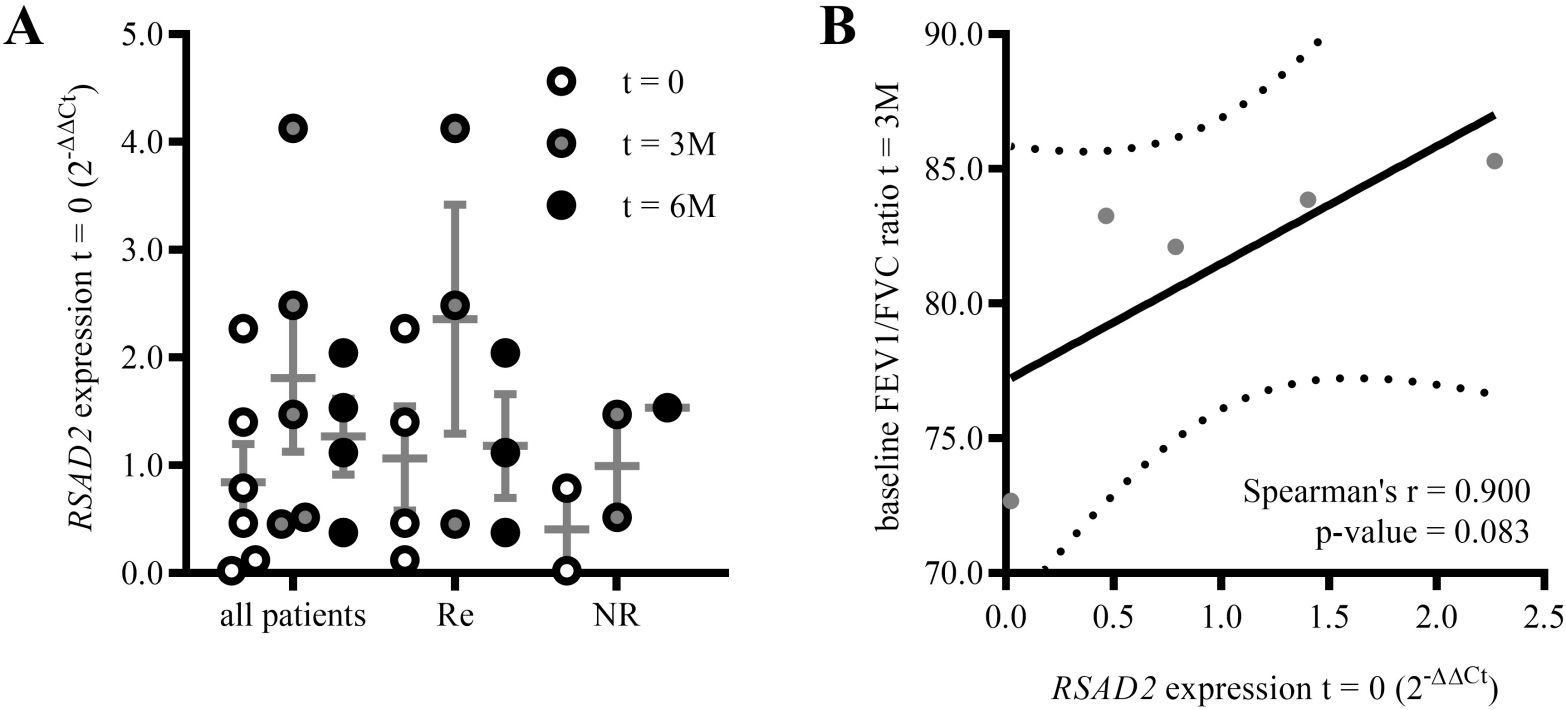
*RSAD2* expression and asthma control in children with severe asthma on omalizumab therapy. **(A)** Expression of *RSAD2* in the total WBC of children with severe asthma responsive (Re) and non-responsive (NR) to omalizumab before induction of the therapy (t=0) and after 3 and 6 months on therapy. **(B)** Spearman’s ranked correlation of *RSAD2* expression in whole WBC of children with severe asthma before the induction of omalizumab therapy (t=0), and baseline FEV1/FVC ratio in the same patients after 3 months of therapy.

To assess the clinical relevance of our results on *RSAD2*, we examined its expression in the WBC of a small clinical cohort- 6 juvenile asthma patients (2 males, 4 females, mean age 13.8 years, GINA steps 2-5, **Supplementary Table S2**) selected to start the omalizumab therapy. Based on a clinical evaluation and improvement as perceived by the patients after 3 and 6 months of therapy, 4 of the patients were assessed as responders and 2 as non-responders to omalizumab. Of the two, one was a clinical non-responder, while the other reported no perceived improvement, and discontinued the omalizumab treatment due to side-effects after the 3 months follow-up. In general, the *RSAD2* expression increased after 3 months of omalizumab therapy compared to the expression before the start of therapy, and stayed increased in the non-responsive group, but not in the responders after 6 months, although the differences were not significant (**Figure 5A**). Of note, the pre-therapy *RSAD2* expression was higher in the responders group compared to the non-responders (not significant).

As an additional exploratory clinical comparison, we also assessed whether the *RSAD2* expression tracked with the available clinical measures. Indeed, the pre-therapy *RSAD2* expression showed a positive, but non-significant, association with the baseline FEV1/FVC ratio (forced expiratory volume in 1 s/forced vital capacity- an indicator of airways obstruction (26)) after 3 months of therapy (Spearman r = 0.900, p = 0.083, **Figure 5B**). No correlation was found between the pre-therapy *RSAD2* expression and the FeNO (fractional exhaled NO) levels, FEV1/FVC ratios or ACT (asthma control test) score at therapy induction (**Supplementary Figures 6A–D**). Similarly, the patients’ GINA step, age at inclusion, sex, comorbidities, and regular therapies besides omalizumab did not influence the pre-therapy *RSAD2* expression (**Supplementary Figures 6E–M**). Taken together, the clinical cohort exhibited *RSAD2* expression trends compatible with those observed in our *in vitro* analyses.

## Discussion

There is a pressing need for improved therapeutic tailoring of biological therapies in pediatric asthma. The clinical characteristics, such as blood eosinophil count, baseline FeNO, FVC and FEV1, are not of much help in this regard. They are either inconclusive, overlap between different therapeutics, or are of questionable relevance for the pediatric population, due to the fact that they were investigated predominantly in adult populations with only a limited number of adolescents included (3, 27–31) Molecular markers thus seem the way forward, but the lack of suitable donors is impeding suitable research. *Ex vivo*/*in vitro* functional cell testing may overcome this issue (11, 13). Here, we have developed an *in vitro* primary cell model to study the cellular and molecular response to omalizumab. Our approach integrates functional testing and cell-type specific high-throughput transcriptomic analysis of the basophils from pediatric/adolescent patients naive to the omalizumab therapy. The proposed *in vitro* model thus offers a dual opportunity- to assess the patients’ response to the omalizumab therapy and to identify potential markers for (non-)response to omalizumab.

Omalizumab binds free serum IgE, which prevents it from binding to the high-affinity receptor FcϵRI, which can be found on the surface of several immune cell subtypes, most prominently on mast cells and basophils (6). Through its action on the latter two, omalizumab reduces histamine release and asthma exacerbations (5). As the mast cells reside in the tissues (*i.e.*, the lungs), *in vitro* functional testing of peripheral blood basophils is a safer and less invasive method to study the patients’ response to omalizumab. The functional part of our *in vitro* model is, thus, based on the basophil activation test (BAT), which estimates the proportion of CD63^++^ basophils in a WBC sample after an allergen challenge. The test is used routinely to confirm allergies to specific allergens, but it was also used, for example, to predict the side-effects of immunotherapy to Hymenoptera venom (32) and anaphylactic reactions to cetuximab treatment (33).

Recently, high basophil reactivity after crosslinking the FcϵRI receptors with rabbit anti-Human IgE polyclonal antibodies was correlated with a poor response of adult severe asthmatics to biological treatments in general (10). Previous reports also showed that basophils isolated from children with a severe asthma/allergy receiving omalizumab treatment for 16 weeks showed reduced activation compared to the ones isolated pre-therapy (9, 34), and that >20 hours of *in vitro* treatment with omalizumab reduced the binding of IgE on the FcϵRI receptors on the basophils (35). Hence, prior to the allergen challenge, for which we used patient-specific allergens, we pre-treated part of each WBC sample with omalizumab for ~24h, to test how this influenced basophil activation in individual patients. The responses to the omalizumab pre-treatment varied between patients. However, besides the expected increase in the % of the CD63^++^ basophils after the allergen challenge and reduced (or not) % of the CD63^++^ basophils in the omalizumab pre-treated samples, we also noticed decreased numbers of total basophils in many of the allergen-challenged samples. These were (partially) counteracted with omalizumab pre-treatment. Using a reverse gating strategy, we determined that part of the CD45^+^CD123^+^HLA-DR^neg^ events (putative basophils) changed the scatter characteristics upon the allergen challenge, and were thus no longer gated as basophils in the experiments. A similar phenomenon was observed before, and attributed to several possible causes, most notably the changed scatter characteristics of some of the basophils due to excessive degranulation, cell death, structural collapse, and also loss/alteration of the marker expression (36). The missing basophils thus represent an activation-associated fraction of basophils under strong stimulation. Therefore, we added them to the % of CD63^++^ basophils, and corrected our classification of the patients as better or poor/non-responders based on this new corrected activity readout.

Compared to the clinical non-response rate, we found too many donors whose basophils did not respond to omalizumab (*i.e.*, the basophil activation after omalizumab treatment decreased minimally or not at all). While it is true that, with a relatively small cohort, each patient has a marked influence on the overall result, there are other possible reasons for discrepancy to consider. Firstly, as explained above, omalizumab can only block free serum IgE binding to unoccupied FcϵRI. The data however show that the majority of the FcϵRI were already occupied by IgE upon testing (**Supplementary Figure S1D**). It is also true that other cells besides basophils (*e.g.*, blood eosinophils and dendritic cells, tissue mast cells) express FcϵRI and/or respond to IgE (5). Thus, we could measure only part of the effect that omalizumab would have in patients over several weeks of therapy (6). Accordingly, our model with short-term omalizumab pre-incubation should not be interpreted as a full representation of the long-term *in vivo* effects of omalizumab, but rather as a partial functional readout. Notably, it was also shown that, already, 1h of treatment with omalizumab resulted in a measurable decrease of the IgE occupied FcϵRI on the basophils *in vitro* (37)! Next, to ensure a reliable activation of the basophils from all the patients, we used rather high, uniform concentrations of the allergens. This might also mask part of a true response to the omalizumab with some patients. For clinical application, titration of the allergen concentration would be prudent before the model readout (38), although this would complicate the logistics of the test markedly.

Regardless of the above limitations for direct clinical applicability, our model still enables investigation of the mechanisms and identification of plausible biomarkers of (non-)response to omalizumab therapy. To this end, we isolated basophils from the WBC samples of the discovery cohort patients, and used them for a whole transcriptome (RNAseq) analysis. In the absence of standardized *in vitro* cut-off values for omalizumab responsiveness, the patients in the discovery cohort were ranked pragmatically and divided into two equal-sized groups based on the BAT Fc values to enable a comparative transcriptomic analysis. We identified several differentially expressed genes when comparing the better and the poor/non-responder groups. Most of these genes showed decreased expression in the poor/non-responder group of patients compared to the better responder group. The gene ontology (GO) analysis revealed a strong association of the downregulated genes with regulation of the viral life cycle and defense response to viral infections. Severe asthmatics are more vulnerable to viral infections, and respiratory viral infections do induce exacerbations, and therefore represent a risk factor for loss of control of allergic asthma (1, 39). Omalizumab therapy, on the other hand, exerts an antiviral effect by stabilizing the effector cells and decreasing the viral shedding (40). Our results now suggest that the low expression of genes connected to the anti-viral response diminishes the protective/stabilizing effect of the omalizumab. The GO analyses thus suggest that the pathways connected to asthma severity are, at the same time, also connected to a poor omalizumab response. Contrary to the majority of clinical studies, our results are based on data from pediatric and juvenile patients- hence, the clinical data from pediatric patients should be investigated more thoroughly either to confirm or reject the above connection.

The four most interesting differentially expressed genes from the RNAseq analysis, all associated with GO terms connected to the defense response to virus- *APOBEC3B*, *HERC5*, *OAS3* and *RSAD2*, were selected for further study in the validation cohort of patients. Again, the basophils were isolated from the WBC samples and used for a targeted expression analysis (qPCR). Given the continuous nature of both molecular and functional readouts we focused primarily on correlation-based analyses. Indeed, we confirmed a significant negative correlation between the *RSAD2* expression in the basophils and the *in vitro* response to omalizumab pre-treatment (*i.e.*, the BAT Fc results), which confirms the RNAseq data. Complementarily, we also made a group-wise comparison. However, when we separated the patients of the validation cohort into the better and poor/non-responder groups, we found no significant differences in expression consistent with the RNAseq data, though *RSAD2* showed a trend toward reduced expression in the poor/non-responder group. Similarly, the exploratory ROC analysis indicated that the baseline *RSAD2* expression in the basophils might have discriminatory potential with respect to the response to omalizumab treatment. Still, given that patient grouping is rank-based rather than defined biologically, and therefore partially arbitrary, and that the limited sample size (particularly of the better responder group) reduces the statistical power and robustness, these results should be interpreted as preliminary, and require further confirmation in larger, independent cohorts. Finally, we also explored the *RSAD2* expression in a small, independent, clinical cohort of pediatric asthmatics starting omalizumab therapy. The baseline *RSAD2* was higher in responders than in the non-responders, and it associated positively with the FEV1/FVC ratio at 3 months of therapy, which is in line with our *in vitro* findings. However, as these findings were not statistically significant, the cohort was very small, and one of the two non-responders discontinued treatment due to intolerance with no perceived improvement rather than clinically confirmed non-response, these observations should be regarded as preliminary and exploratory only.

RSAD2 (Radical S-Adenosyl Methionine Domain-containing 2), also known as viperine, is an interferon-inducible protein that inhibits viral replication and modulates immune responses via Toll-like receptors and interferons (41, 42). An increase in the *RSAD2* expression has been associated with a protective response against viral infections, such as respiratory syncytial virus and rhinovirus, which have the potential to trigger asthma exacerbations (43, 44). Further, FGF10 attenuated the allergic airway inflammation via inhibiting the PI3K/AKT/NF-κB pathway and upregulated the *RSAD2* expression in the lung tissue of a murine asthma model (45). This is consistent with our findings, where high *RSAD2* expression is associated with better response to omalizumab, and may be associated with better asthma control. On the other hand, *RSAD2* knockdown reduced inflammation and Th2 cell differentiation from naive CD4^+^ T-cells in another murine asthma model, which is contrary to the above report and our results (46). Our findings indicate an association between *RSAD2* expression and response to omalizumab, which is probably not reflecting a direct mechanistic interaction though. Rather, the *RSAD2* expression may reflect baseline immune or interferon-related states that influence the responsiveness to omalizumab. A low *RSAD2* expression is associated with a diminished response to viral infections, which, in turn, leads to increased asthma exacerbations and poorer asthma control (47, 48). The presented *in vitro* model to study and predict the response of severe asthma patients to omalizumab therapy thus provides clinically relevant outcomes. Some limitations and potential refinements discussed above should, however, be considered, and the model evaluated on patients scheduled to receive omalizumab therapy. Despite the consistency of the results across three independent albeit relatively small, patient cohorts, the potential role of *RSAD2* as a predictive biomarker of response to omalizumab requires further validation in larger clinical settings, and with additional clinical indicators of lung function and asthma control before it can be considered generalizable. Further studies are also needed to elucidate the biological pathways linking *RSAD2* expression to variability in response to omalizumab.

The presented *in vitro* model provides a dual opportunity. It is a potential method for predicting the response to omalizumab prior to the actual treatment, thus also reducing the costs of trial-and-error therapy for the patients and healthcare. In addition, it serves as a platform to explore the mechanisms underlying non-responsiveness to omalizumab and to identify potential biomarkers of non-response. Using the above model, we demonstrated *RSAD2* expression in the whole blood and in basophils as a potential predictive biomarker of response to omalizumab therapy in pediatric asthma. Further, we provided data showing that the molecular pathways connected with defense against viral infections influence the differential response to omalizumab treatment. This exploration could potentially pave the way for the development of complementary therapeutic strategies, and represents a holistic and promising approach in the effort to improve the treatment landscape for pediatric asthma.

## Data Availability

The datasets presented in this study can be found in online repositories. The names of the repository/repositories and accession number(s) can be found below: https://www.ncbi.nlm.nih.gov/geo/, GSE281339.
