# Supplementary material for: A functional cell model using basophil activation test to study molecular mechanisms and biomarkers of response to omalizumab treatment in patients with asthma
Source: Front Immunol. 2026 Feb 27;17:1744735. doi: 10.3389/fimmu.2026.1744735 (PMC12982396; doi:10.3389/fimmu.2026.1744735)
Supplement: Supplementary file 1 [file DataSheet1.pdf]

Supplementary Material

Supplementary Figures:

Supplementary Figure S1: The basophil activation test model.

Supplementary Figure S2: Partial cytolysis of basophils after exposure to allergens.

Supplementary Figure S3: Relations between BAT Fc results and basic characteristics of patients/samples in discovery cohort.

Supplementary Figure S4: BAT results and their relations to basic characteristics of patients/samples in the validation cohort.

Supplementary Figure S5: Group-wise analysis of *RSAD2* expression in the validation cohort.

Supplementary Figure S6: Relations between *RSAD2* expression and basic characteristics of patients in the clinical cohort.

Supplementary Tables:

Supplementary Table ST1: Patients' data - Discovery and Validation Cohorts.

Supplementary Table ST2: Patients' data - Clinical Cohort.

Supplementary Table ST3: qRT-PCR primers.

Supplementary Table ST4: Differentially expressed genes of the basophils from the better and poorer/non-responder group of patients ($|\log_2 FC|>2.0$, $p<0.05$).

Supplementary Table ST5: Over-represented GO terms associated with genes differentially expressed between the better and poorer/non-responder group after adjustment for multiple testing ($|\log_2 FC|>2.0$, adjusted $p<0.05$).

Supplementary Table ST6: Exploratory ROC performance metrics for baseline (no treatment) *RSAD2* expression in the validation cohort.

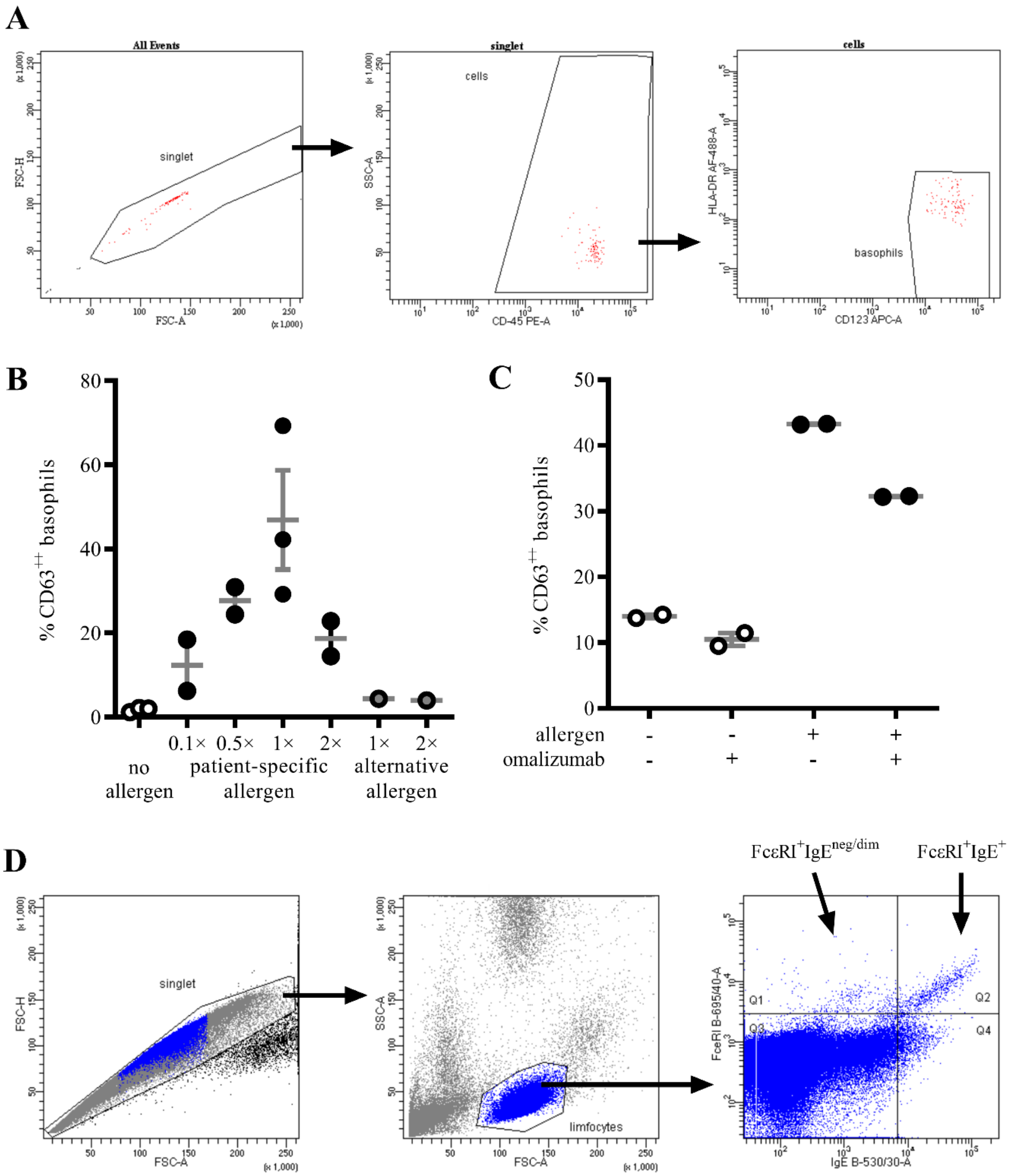

Supplementary Figure S1: The basophil activation test model. **(A)** Purity control of FACS isolated basophils ($CD45^{+}CD123^{+}HLA\text{-}DR^{neg}$). The corresponding unsorted white blood cells (WBC) sample is shown in Figure 1. **(B)** % of the $CD63^{++}$ basophils in WBC samples after exposure to patient-specific and alternative allergens. The alternative allergen was used in normal test and excessive (2×) concentration. **(C)** % of the $CD63^{++}$ basophils in untreated and omalizumab treated WBC samples after exposure to the patient-specific allergen. **(D)** Fresh WBC were stained for FcεRI (Mouse anti-Human FcεRI-PerCP-eFluor710, Invitrogen 46-5899-42) and IgE (Rabbit anti-Human IgE, Dako A0094, followed by Goat anti-Rabbit IgG-Alexa 488, Abcam ab150077). FSC-A/SSC-A dot plot was used to gate for lymphocytes (the gate includes basophils). The majority of the FcεRI expressing cells is highly positive for IgE (Q2), but a portion of these cells is IgE negative/dim (Q1).

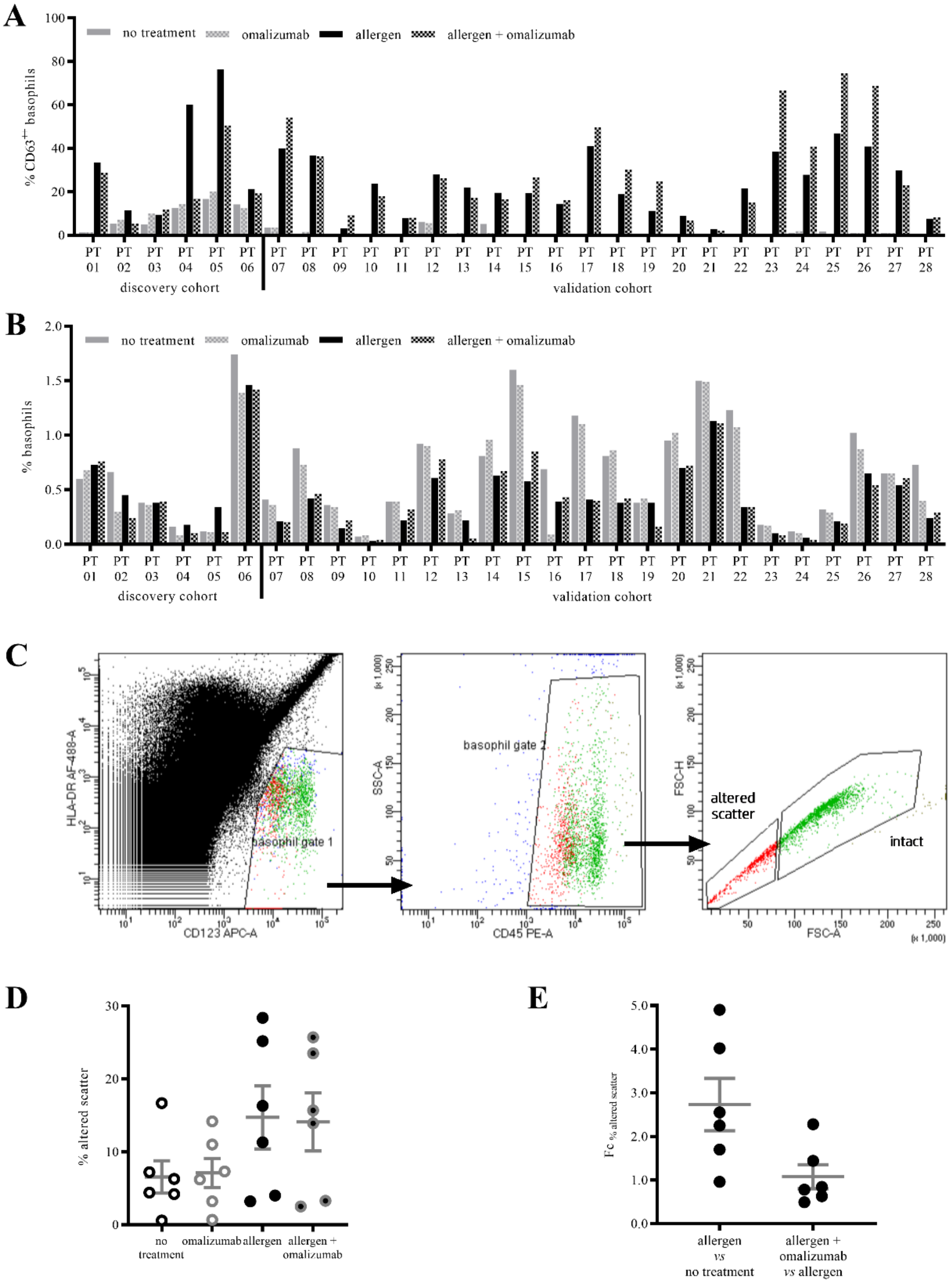

Supplementary Figure S2: Partial cytolysis of basophils after exposure to allergens. **(A)** % of the $CD63^{++}$ basophils in control (no treatment) and omalizumab pre-treated WBC samples after exposure to the patient-specific allergens. **(B)** % of total basophils in untreated (control) and omalizumab treated WBC samples after exposure to the patient-specific allergens. **(C)** Reverse gating: total WBC were gated for $CD45^{+}CD123^{+}$HLA-DR^{neg} events (i.e., basophils). Next the gated events were separated into intact single cells and altered scatter events based on FSC-A/FSC-H ratio. **(D)** Quantification of altered scatter events in the $CD45^{+}CD123^{+}$HLA-DR^{neg} population after exposure to allergen and omalizumab treatment. **(E)** Fold changes in % of altered scatter events in the $CD45^{+}CD123^{+}$HLA-DR^{neg} population after exposure to allergen and omalizumab treatment.

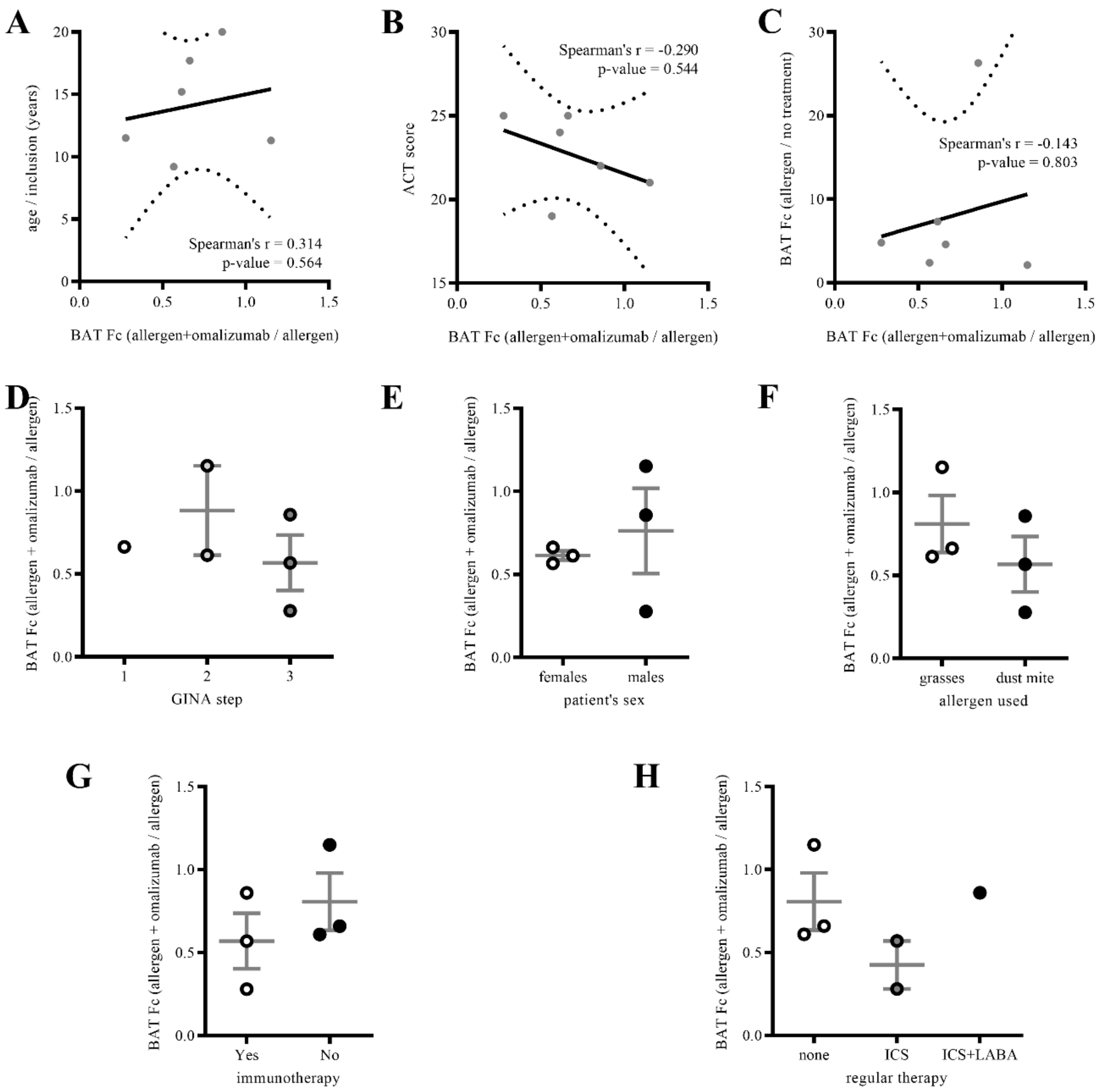

Supplementary Figure S3: Relations between BAT Fc results and basic characteristics of patients/samples in discovery cohort. **(A-C)** Dot plots showing correlation of the BAT Fc results (allergen + omalizumab / allergen) to **(A)** patients' age at inclusion, **(B)** patients' ACT score at inclusion, **(C)** fold-change in activated basophils after allergen challenge (BAT Fc allergen / no treatment). **(D-H)** Scatter plots showing differences in the BAT Fc results between **(D)** patients with different GINA steps, **(E)** samples from female and male patients, **(F)** samples treated with different allergens, **(G)** samples from patients receiving immunotherapy or not, **(H)** samples from patients receiving different regular therapy for asthma (ICS-inhaled corticosteroid, ICS+LABA- combination of ICS and long-acting β adrenoceptor agonist) or not.

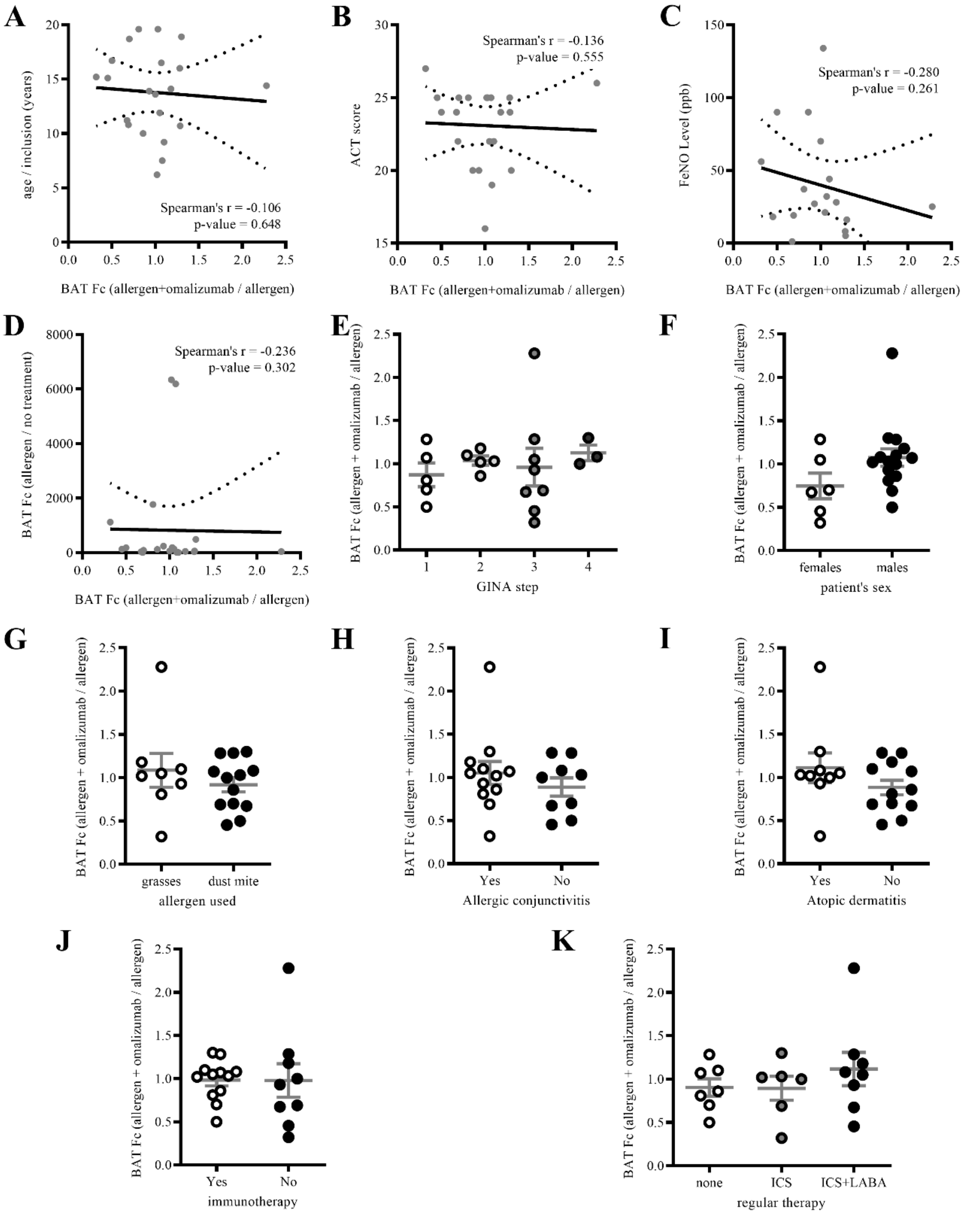

Supplementary Figure S4: BAT results and their relations to basic characteristics of patients/samples in the validation cohort. **(A-D)** Dot plots showing correlation of the BAT Fc results (allergen + omalizumab / allergen) to **(A)** patients' age at inclusion, **(B)** patients' ACT score at inclusion, **(C)** patients' FeNO level at inclusion, **(D)** fold-change in activated basophils after allergen challenge (BAT Fc allergen / no treatment). **(E-K)** Scatter plots showing differences in the BAT Fc results between **(E)** patients with different GINA steps, **(F)** samples from female and male patients, **(G)** samples treated with different allergens, **(H)** samples from patients with/without allergic conjunctivitis, **(I)** samples from patients with/without atopic dermatitis, **(J)** samples from patients receiving immunotherapy or not, **(K)** samples from patients receiving different regular therapy for asthma (ICS- inhaled corticosteroid, ICS+LABA- combination of ICS and long-acting β adrenoceptor agonist) or not.

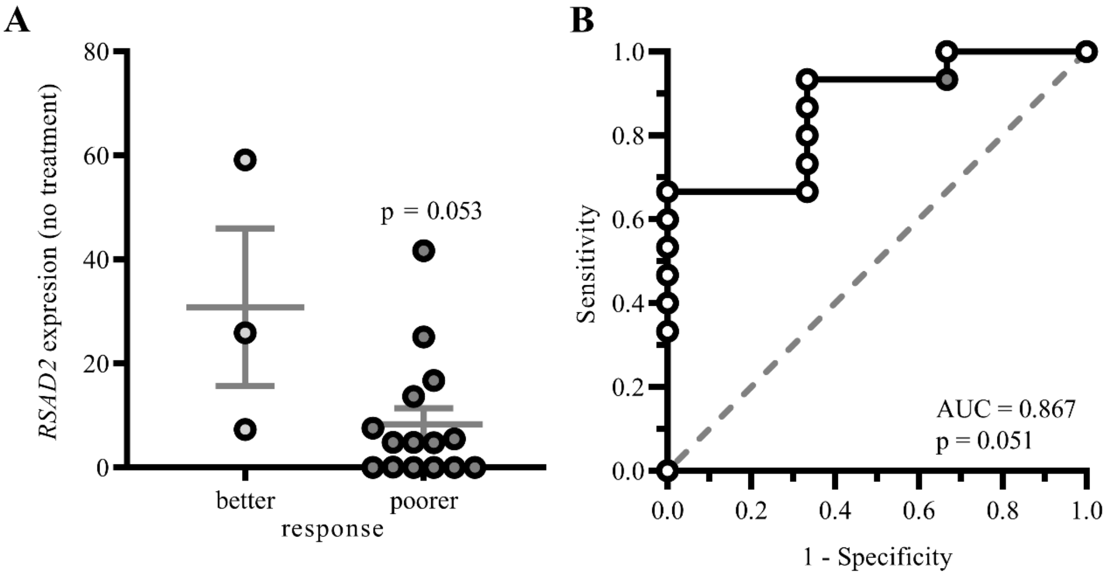

Supplementary Figure S5: Group-wise analysis of *RSAD2* expression in the validation cohort. **(A)** Baseline (no treatment) *RSAD2* expression in the basophils of better and poorer responders to omalizumab (as determined by *in vitro* testing). **(B)** Receiver operating characteristic (ROC) curve showing the ability of baseline *RSAD2* expression in basophils to discriminate between better and poorer responders to omalizumab (as determined by *in vitro* testing).

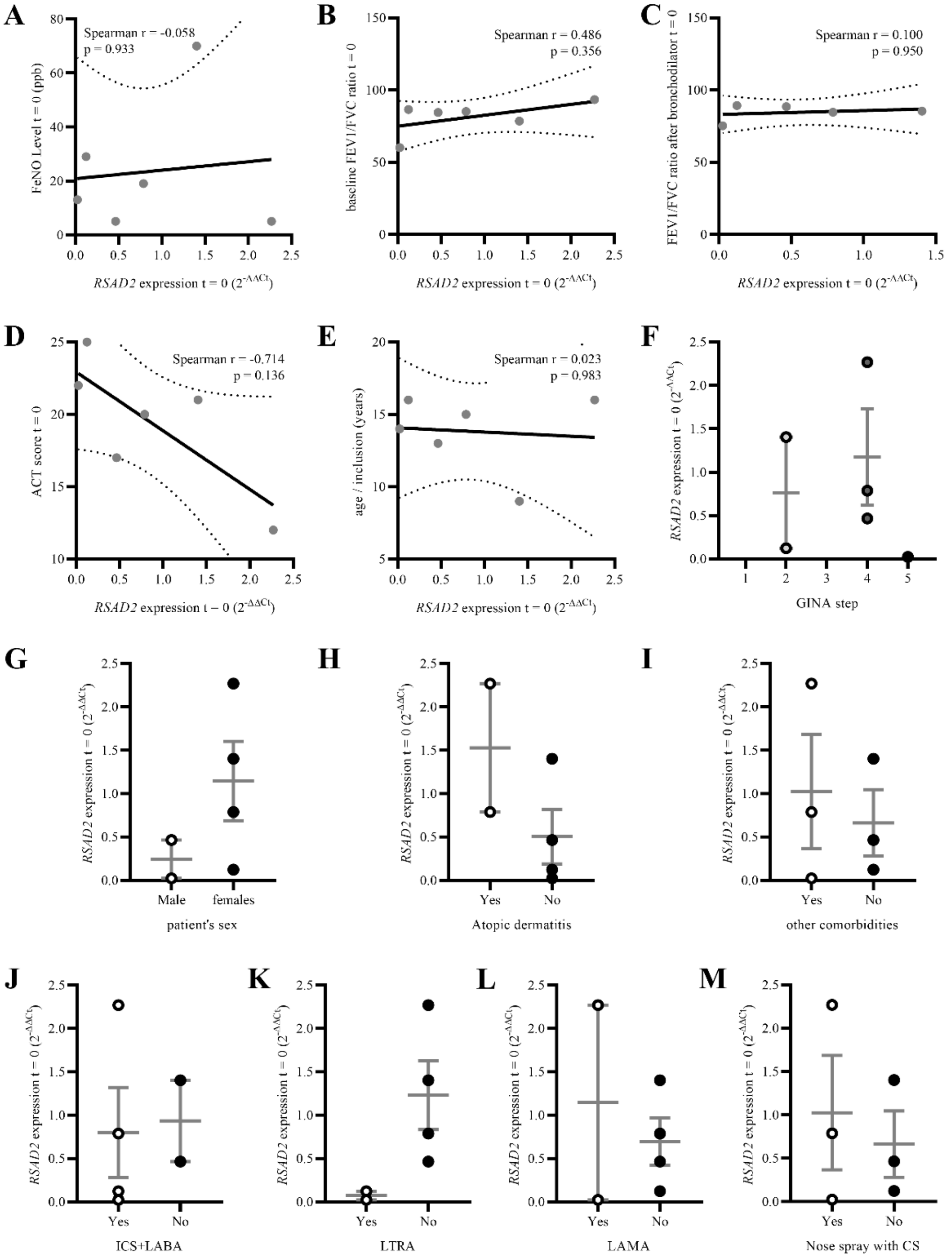

Supplementary Figure S6: Relations between *RSAD2* expression and basic characteristics of patients in the clinical cohort. **(A-E)** Dot plots showing correlation of the *RSAD2* expression in whole WBC of children with severe asthma before induction of omalizumab therapy (t=0) and **(A)** patients' FeNO level at inclusion, **(B)** patients' baseline FEV/FVC1 ratio at inclusion, **(C)** patients' FEV/FVC1 ratio after bronchodilator test at inclusion, **(D)** patients' ACT score at inclusion, **(E)** patients' age at inclusion. **(F-M)** Scatter plots showing differences in the *RSAD2* expression in whole WBC of children with severe asthma before induction of omalizumab therapy (t=0) between **(F)** patients with different GINA steps, **(G)** female and male patients, **(H)** patients with/without atopic dermatitis, **(I)** patients with/without other comorbidities, **(J)** patients receiving combination of inhaled corticosteroid and long-acting β adrenoceptor agonist (ICS+LABA) or not, **(K)** patients receiving leukotriene receptor antagonist (LTRA) treatment or not, **(L)** patients receiving long-acting muscarinic antagonist (LAMA) treatment or not, **(M)** patients using nose spray with corticosteroids (CS) or not.

Supplementary Table ST1: Patients' data - Discovery and Validation Cohorts.

Patient	Sex	Age at incl.	Latest GINA step	ACT score at incl.	FeNO level at incl.	Latest IgE level (ng/mL)	Diagnosed allergic diseases	Other comorbidities	Regular medication	Other current medications	Allergies
Discovery Cohort (Country of origin: Slovenia)											
PT01	M	20.0	3	22	70 ppb	n.d.	AC, AR	/	ICS+LABA	immuno.	HDM, gr., othr.
PT02	F	15.2	2	24	141 ppb	n.d.	AR, AD	/	/	/	HDM, gr., othr.
PT03	M	11.3	2	21	64 ppb	33	AC, AR	/	/	/	HDM, gr., othr.
PT04	M	11.5	3	25	n.d.	n.d.	AR	/	ICS	immuno.	HDM
PT05	F	17.7	1	25	n.d.	n.d.	AC, AR	GR, psych.	/	/	gr., othr.
PT06	F	9.2	3	19	n.d.	298	AC, AR	GR	ICS	immuno.	HDM, gr., othr.
Validation Cohort (Country of origin: Slovenia)											
PT07	M	7.5	4	19	n.d.	322	AR, AD	/	ICS+LABA	NCS, immuno.	HDM, gr., othr.
PT08	M	10.0	2	20	90 ppb	n.d.	AC, AR	/	/	immuno.	HDM, gr., othr.
PT09	F	18.7	1	25	n.d.	n.d.	AR	/	/	immuno.	HDM, gr., othr.
PT10	M	6.2	2	25	n.d.	n.d.	AC, AR, AD	/	ICS	immuno.	gr., othr.
PT11	M	16.7	1	24	90 ppb	n.d.	AR	/	/	immuno.	HDM, gr., othr.
PT12	M	10.8	3	22	19 ppb	n.d.	AC, AR	/	ICS	/	HDM
PT13	M	14.4	3	26	25 ppb	n.d.	AC, AR, AD	/	ICS+LABA	/	HDM, gr., othr.
PT14	M	9.2	2	22	44 ppb	n.d.	AC, AR	/	/	immuno.	gr., othr.
PT15	M	19.6	1	25	37 ppb	n.d.	AC, AR	/	/	immuno.	HDM, gr., othr.
PT16	F	15.2	3	27	56 ppb	n.d.	AC, AR, AD	/	ICS, LTRA	/	gr., othr.
PT17	M	19.6	2	25	134 ppb	410	AR, AD	/	ICS	immuno.	HDM, othr.
PT18	M	16.5	1	25	32 ppb	125	AC, AR	/	/	immuno.	HDM, othr.
PT19	M	12.7	4	22	8 ppb	n.d.	AC, AR	/	ICS+LABA	a-hist., NCS	HDM, gr., othr.
PT20	F	11.9	3	22	21 ppb	n.d.	AC, AR, AD	GR	ICS	immuno.	gr., othr.
PT21	M	13.6	4	16	70 ppb	n.d.	AR, AD	/	ICS+LABA	a-hist., NCS	HDM, othr.
PT22	M	13.9	3	20	27 ppb	n.d.	AC, AR, AD	/	ICS	/	gr., othr.
PT23	M	18.9	4	20	16 ppb	n.d.	AC, AR, AD	LI	ICS+LABA	immuno.	HDM, gr., othr.
PT24	M	14.1	2	24	28 ppb	n.d.	AC, AR	/	ICS	/	HDM, gr., othr.
PT25	M	16.0	1	25	8 ppb	213	AR	/	/	immuno.	HDM
PT26	F	10.7	3	24	5 ppb	1314	/	/	ICS+LABA	/	HDM, othr.
PT27	F	11.2	3	24	1 ppb	n.d.	AR	/	ICS+LABA	/	HDM
PT28	F	15.1	3	25	18 ppb	642	AR	/	ICS+LABA	/	HDM, othr.

General abbreviations: F (female), M (male); incl. (inclusion); n.d. (no data); **Diseases:** AC (allergic conjunctivitis), AR (allergic rhinitis), AD (atopic dermatitis), GR (gastroesophageal reflux), LI (lactose intolerance), psych. (psychological factors); **Therapies:** a-hist. (antihistaminic); ICS (inhaled corticosteroid); immuno. (immunotherapy); LABA (Long acting β adrenoceptor agonist); LTRA (leukotriene receptor antagonist); NCS (nose spray with corticosteroid); **Allergies:** HDM (house dust mite); gr. (grasses); othr. (other allergens).

Supplementary Table ST2: Patients' data - Clinical Cohort.

Patient	Sex	Age at incl.	Latest GINA step	ACT score at incl.	FeNO level at incl.	Latest IgE level (ng/mL)	Diagnosed allergic diseases	Other comorbidities	Regular medication	Other current medications	Allergies	Clinical response to oma.
Clinical Cohort (Country of origin: Sweden)												
CPT01	F	9	2	21	70 ppb	n.d.	AC, AR		ICS, oma.	a-hist.	othr.	Yes
CPT02	F	15	4	20	19 ppb	5700	AC, AR, AD	psych.	ICS+LABA, oma.	a-hist., NCS	gr., othr.	No
CPT03	M	13	4	17	5 ppb	410	AC, AR		oma.	a-hist.	HDM, gr., othr.	Yes
CPT04	F	16	4	12	5 ppb	n.d.	AR, AD, CU	bronchiectasis	LAMA, ICS+LABA, oma.	NCS	othr.	Yes
CPT05	M	14	5	22	13 ppb	200	AC, AR	obesity	LAMA, ICS+LABA, LTRA, oma.	a-hist., NCS	HDM, othr.	No
CPT06	F	16	2	25	29 ppb	4200	AC, AR		ICS+LABA, LTRA, oma.	a-hist.	HDM, gr., othr.	Yes

General abbreviations: F (female), M (male); Clin. (clinical); incl. (inclusion); n.d. (no data); **Diseases:** AC (allergic conjunctivitis), AR (allergic rhinitis), AD (atopic dermatitis), CU (chronic urticaria), psych. (psychological factors); **Therapies:** a-hist. (antihistaminic); ICS (inhaled corticosteroid); LABA (Long acting β adrenoceptor agonist); LAMA (long-acting muscarinic antagonist); LTRA (leukotriene receptor antagonist); NCS (nose spray with corticosteroid); oma. (omalizumab); **Allergies:** HDM (house dust mite); gr. (grasses); othr. (other allergens).

Supplementary Table ST3: qRT-PCR primers.

Gene Symbol	Gene ID	Annealing T (°C)	Forward Primer (5'→3')	Reverse Primer (5'→3')
APOBEC3B	9582	58.1	CATCCTCTATGGTCGGAGC	TTTCTGCGTGGTACTGAGGC
HERC5	51191	65.7	TTTCCCTCAACCACCACACC	ATCTTTACTCTCAGTGTGGCCC
OAS3	4940	65.7	TGAAAACTGTCAAGGGAGGCT	ATCTACGGATGTCAGGCGGA
RSAD2	91543	60.0	GTGGAAGAGGACATGACGGA	CAGAAAGCGCATATATTCATCCA
B2M	567	60.0	TTCTGGCCTGGAGGCTATC	TCAGGAAATTTGACTTTCCATTC
GAPDH	2597	60.0	TGAGAACGGGAAGCTTGTCA	CCCTGCAAATGAGCCCCA
RNA18SN5	100008588	60.0	GCAATTATTCCCCATGAACG	GGGACTTAATCAACGCAAGC

Supplementary Table ST4: Differentially expressed genes of the basophils from the better and poorer/non-responder group of patients ($|log_2FC|>2.0$, $p<0.05$).

Gene Symbol	Gene ID	Gene Name	log_2FC	p-value	adj. p-value
OAS3	**4940**	**2'-5'-oligoadenylate synthetase 3**	**-3.558**	**2.336E-07**	**1.293E-03**
DUSP2	1844	dual specificity phosphatase 2	-2.030	3.360E-07	1.293E-03
HLA-C	3107	major histocompatibility complex; class I; C	-4.100	4.568E-07	1.318E-03
EPSTI1	94240	epithelial stromal interaction 1	-3.354	1.952E-06	4.195E-03
HERC5	**51191**	**HECT and RLD domain containing E3 ubiquitin protein ligase 5**	**-2.508**	**2.956E-06**	**4.195E-03**
CMPK2	129607	cytidine/uridine monophosphate kinase 2	-3.194	3.442E-06	4.195E-03
RSAD2	**91543**	**radical S-adenosyl methionine domain containing 2**	**-3.993**	**4.762E-06**	**4.582E-03**
APOBEC3B	**9582**	**apolipoprotein B mRNA editing enzyme catalytic subunit 3B**	**-2.786**	**5.107E-05**	**2.963E-02**
CCDC163	126661	coiled-coil domain containing 163	-2.165	5.133E-05	2.963E-02
HDC	3067	histidine decarboxylase	-2.393	4.465E-04	8.165E-02
IFI44L	10964	interferon induced protein 44 like	-2.781	6.399E-04	9.850E-02
H2BC5	3017	H2B clustered histone 5	-2.468	6.796E-04	1.006E-01
IFIT1	3434	interferon induced protein with tetratricopeptide repeats 1	-3.519	8.075E-04	1.072E-01
EIF2AK2	5610	eukaryotic translation initiation factor 2 alpha kinase 2	-2.492	1.022E-03	1.203E-01
HBB	3043	hemoglobin subunit beta	5.678	1.351E-03	1.335E-01
ZCCHC2	54877	zinc finger CCHC-type containing 2	-2.562	1.949E-03	1.510E-01
IL1B	3553	interleukin 1 beta	-3.481	2.367E-03	1.617E-01
IFI44	10561	interferon induced protein 44	-2.077	2.810E-03	1.751E-01
MYL9	10398	myosin light chain 9	2.678	3.225E-03	1.881E-01
BEX3	27018	brain expressed X-linked 3	2.355	3.405E-03	1.881E-01
GBP5	115362	guanylate binding protein 5	-2.784	3.432E-03	1.881E-01
IFIT3	3437	interferon induced protein with tetratricopeptide repeats 3	-4.450	4.303E-03	2.001E-01
HBA1	3039	hemoglobin subunit alpha 1	5.428	4.551E-03	2.024E-01
HBA2	3040	hemoglobin subunit alpha 2	5.376	5.097E-03	2.094E-01
TNFAIP3	7128	TNF alpha induced protein 3	-2.795	6.809E-03	2.464E-01
MAP3K7CL	56911	MAP3K7 C-terminal like	2.773	7.636E-03	2.505E-01
GBP1	2633	guanylate binding protein 1	-2.113	7.981E-03	2.541E-01
SERPING1	710	serpin family G member 1	-2.533	8.297E-03	2.558E-01
OAS1	4938	2'-5'-oligoadenylate synthetase 1	-2.945	1.020E-02	2.671E-01
TNFSF10	8743	TNF superfamily member 10	-2.110	1.117E-02	2.757E-01
H2AC19	723790	H2A clustered histone 19	-2.758	1.269E-02	2.920E-01
TAGAP	117289	T cell activation Rho GTPase activating protein	-2.170	1.316E-02	2.939E-01
OSM	5008	oncostatin M	-3.884	1.317E-02	2.939E-01
PPP1R15A	23645	protein phosphatase 1 regulatory subunit 15A	-3.218	1.327E-02	2.940E-01
ZFP36	7538	ZFP36 ring finger protein	-2.471	1.423E-02	2.989E-01

Gene Symbol	Gene ID	Gene Name	log_2FC	p-value	adj. p-value
TRDV2	28517	T cell receptor delta variable 2	-2.329	1.464E-02	3.008E-01
CAVIN2	8436	caveolae associated protein 2	2.466	1.473E-02	3.008E-01
FOS	2353	Fos proto-oncogene; AP-1 transcription factor subunit	-3.413	1.648E-02	3.100E-01
BTG2	7832	BTG anti-proliferation factor 2	-2.719	1.748E-02	3.164E-01
IFIT2	3433	interferon induced protein with tetratricopeptide repeats 2	-3.915	1.757E-02	3.166E-01
SAT1	6303	spermidine/spermine N1-acetyltransferase 1	-2.201	1.813E-02	3.201E-01
DUSP1	1843	dual specificity phosphatase 1	-2.903	1.833E-02	3.206E-01
NFKBIA	4792	NFKB inhibitor alpha	-2.432	1.844E-02	3.206E-01
MT-ND3	4537	mitochondrially encoded NADH dehydrogenase 3	2.025	1.976E-02	3.227E-01
CD274	29126	CD274 molecule	-2.051	2.131E-02	3.253E-01
KCNJ2	3759	potassium inwardly rectifying channel subfamily J member 2	-2.574	2.327E-02	3.322E-01
IL1RN	3557	interleukin 1 receptor antagonist	-2.402	2.393E-02	3.334E-01
NRGN	4900	neurogranin	2.318	2.429E-02	3.363E-01
H2AC18	8337	H2A clustered histone 18	-2.505	2.548E-02	3.417E-01
EPHB1	2047	EPH receptor B1	-2.089	2.657E-02	3.467E-01
LOC100505479	100505479	hypothetical LOC100505479	2.130	2.754E-02	3.491E-01
GNG11	2791	G protein subunit gamma 11	2.338	2.869E-02	3.503E-01
RN7SK	125050	RNA component of 7SK nuclear ribonucleoprotein	2.897	2.932E-02	3.503E-01
LOC100506979	100506979	hypothetical LOC100506979	-2.653	2.969E-02	3.508E-01
IER5L	389792	immediate early response 5 like	2.007	3.001E-02	3.528E-01
H2BC21	8349	H2B clustered histone 21	-2.657	3.065E-02	3.565E-01
PF4	5196	platelet factor 4	2.891	3.152E-02	3.599E-01
DNAJB1	3337	DnaJ heat shock protein family (Hsp40) member B1	-2.344	3.261E-02	3.638E-01
SOCS3	9021	suppressor of cytokine signaling 3	-2.158	3.265E-02	3.638E-01
FAS	355	Fas cell surface death receptor	-2.081	3.268E-02	3.638E-01
TREML1	340205	triggering receptor expressed on myeloid cells like 1	2.099	3.331E-02	3.666E-01
NR4A2	4929	nuclear receptor subfamily 4 group A member 2	-3.487	3.516E-02	3.765E-01
DHRS9	10170	dehydrogenase/reductase 9	-3.009	3.592E-02	3.791E-01
CXCL1	2919	C-X-C motif chemokine ligand 1	-3.228	3.693E-02	3.815E-01
CLU	1191	clusterin	2.475	3.987E-02	3.905E-01
ICAM1	3383	intercellular adhesion molecule 1	-2.002	4.067E-02	3.923E-01
MT-RNR1	4549	mitochondrially encoded 12S RNA	2.203	4.202E-02	3.952E-01
HSPA1A	3303	heat shock protein family A (Hsp70) member 1A	-3.046	4.207E-02	3.952E-01
REL	5966	REL proto-oncogene; NF-kB subunit	-2.074	4.217E-02	3.955E-01
MT-ND5	4540	mitochondrially encoded NADH dehydrogenase 5	2.207	4.510E-02	4.042E-01
BATF2	116071	basic leucine zipper ATF-like transcription factor 2	-2.549	4.778E-02	4.109E-01

Supplementary Table ST5: Over-represented GO terms associated with genes differentially expressed between the better and poorer/non-responder group after adjustment for multiple testing ($|log_2FC|>2.0$, adjusted $p<0.05$).

GO term ID	GO Term	Fold Enrichment	Enrichment FDR	Associated Genes Found
GO:0045071	Negative regulation of viral genome replication	83	0.0013	*OAS3, APOBEC3B, RSAD2*
GO:0045069	Regulation of viral genome replication	51	0.0020	*OAS3, APOBEC3B, RSAD2*
GO:0048525	Negative regulation of viral process	49	0.0020	*OAS3, APOBEC3B, RSAD2*
GO:0019079	Viral genome replication	34	0.0032	*OAS3, APOBEC3B, RSAD2*
GO:1903900	Regulation of viral life cycle	33	0.0032	*OAS3, APOBEC3B, RSAD2*
GO:0050792	Regulation of viral process	27	0.0046	*OAS3, APOBEC3B, RSAD2*
GO:0051607	Defense response to virus	19	0.0020	*OAS3, RSAD2, APOBEC3B, HERC5*
GO:0009615	Response to virus	15	0.0032	*OAS3, RSAD2, APOBEC3B, HERC5*
GO:0071659	Negative regulation of IP-10 production	396	0.0300	*OAS3*
GO:0071612	IP-10 production	297	0.0300	*OAS3*
GO:0071650	Negative regulation of chemokine (C-C motif) ligand 5 production	297	0.0300	*OAS3*
GO:0071658	Regulation of IP-10 production	297	0.0300	*OAS3*
GO:2000342	Negative regulation of chemokine (C-X-C motif) ligand 2 production	297	0.0300	*OAS3*
GO:0006235	dTTP biosynthetic process	297	0.0300	*CMPK2*
GO:0009189	Deoxyribonucleoside diphosphate biosynthetic process	297	0.0300	*CMPK2*
GO:0009212	Pyrimidine deoxyribonucleoside triphosphate biosynthetic process	297	0.0300	*CMPK2*
GO:0046075	dTTP metabolic process	297	0.0300	*CMPK2*
GO:0044355	Clearance of foreign intracellular DNA	297	0.0300	*APOBEC3B*
GO:0099046	Clearance of foreign intracellular nucleic acids	297	0.0300	*APOBEC3B*
GO:0034157	Positive regulation of toll-like receptor 7 signaling pathway	237	0.0300	*RSAD2*
GO:2000553	Positive regulation of T-helper 2 cell cytokine production	237	0.0300	*RSAD2*
GO:0009186	Deoxyribonucleoside diphosphate metabolic process	237	0.0300	*CMPK2*
GO:0034155	Regulation of toll-like receptor 7 signaling pathway	198	0.0300	*RSAD2*
GO:0035745	T-helper 2 cell cytokine production	198	0.0300	*RSAD2*
GO:2000551	Regulation of T-helper 2 cell cytokine production	198	0.0300	*RSAD2*
GO:0060700	Regulation of ribonuclease activity	198	0.0300	*OAS3*
GO:0002486	Antigen processing and presentation of endogenous peptide antigen via MHC class I via ER pathway TAP-independent	198	0.0300	*HLA-C*
GO:0032020	ISG15-protein conjugation	198	0.0300	*HERC5*
GO:0009139	Pyrimidine nucleoside diphosphate biosynthetic process	198	0.0300	*CMPK2*
GO:0009211	Pyrimidine deoxyribonucleoside triphosphate metabolic process	198	0.0300	*CMPK2*
GO:0009221	Pyrimidine deoxyribonucleotide biosynthetic process	198	0.0300	*CMPK2*
GO:0016554	Cytidine to uridine editing	198	0.0300	*APOBEC3B*
GO:0070383	DNA cytosine deamination	198	0.0300	*APOBEC3B*

GO term ID	GO Term	Fold Enrichment	Enrichment FDR	Associated Genes Found
GO:0034165	Positive regulation of toll-like receptor 9 signaling pathway	170	0.0300	*RSAD2*
GO:0071609	Chemokine (C-C motif) ligand 5 production	170	0.0300	*OAS3*
GO:0071649	Regulation of chemokine (C-C motif) ligand 5 production	170	0.0300	*OAS3*
GO:0002476	Antigen processing and presentation of endogenous peptide antigen via MHC class Iβ	170	0.0300	*HLA-C*
GO:0002484	Antigen processing and presentation of endogenous peptide antigen via MHC class I via ER pathway	170	0.0300	*HLA-C*
GO:0009138	Pyrimidine nucleoside diphosphate metabolic process	170	0.0300	*CMPK2*
GO:0046940	Nucleoside monophosphate phosphorylation	170	0.0300	*CMPK2*
GO:0002428	Antigen processing and presentation of peptide antigen via MHC class Iβ	148	0.0324	*HLA-C*
GO:0016553	Base conversion or substitution editing	132	0.0357	*APOBEC3B*
GO:0002830	Positive regulation of type 2 immune response	119	0.0364	*RSAD2*
GO:0035743	CD4-positive alpha-beta T cell cytokine production	119	0.0364	*RSAD2*
GO:0039530	MDA-5 signaling pathway	119	0.0364	*OAS3*
GO:0045006	DNA deamination	119	0.0364	*APOBEC3B*
GO:0034154	Toll-like receptor 7 signaling pathway	108	0.0375	*RSAD2*
GO:0071639	Positive regulation of monocyte chemotactic protein-1 production	108	0.0375	*OAS3*
GO:0009133	Nucleoside diphosphate biosynthetic process	108	0.0375	*CMPK2*
GO:0034163	Regulation of toll-like receptor 9 signaling pathway	99	0.0375	*RSAD2*
GO:0002475	Antigen processing and presentation via MHC class Iβ	99	0.0375	*HLA-C*
GO:0045869	Negative regulation of single stranded viral RNA replication via double stranded DNA intermediate	99	0.0375	*APOBEC3B*
GO:0019885	Antigen processing and presentation of endogenous peptide antigen via MHC class I	91	0.0399	*HLA-C*
GO:0072567	Chemokine (C-X-C motif) ligand 2 production	85	0.0401	*OAS3*
GO:2000341	Regulation of chemokine (C-X-C motif) ligand 2 production	85	0.0401	*OAS3*
GO:0009263	Deoxyribonucleotide biosynthetic process	85	0.0401	*CMPK2*
GO:0009265	2-deoxyribonucleotide biosynthetic process	85	0.0401	*CMPK2*
GO:0046385	Deoxyribose phosphate biosynthetic process	85	0.0401	*CMPK2*
GO:0071605	Monocyte chemotactic protein-1 production	79	0.0408	*OAS3*
GO:0071637	Regulation of monocyte chemotactic protein-1 production	79	0.0408	*OAS3*
GO:0002483	Antigen processing and presentation of endogenous peptide antigen	79	0.0408	*HLA-C*
GO:0045091	Regulation of single stranded viral RNA replication via double stranded DNA intermediate	79	0.0408	*APOBEC3B*
GO:0032682	Negative regulation of chemokine production	74	0.0411	*OAS3*
GO:0009148	Pyrimidine nucleoside triphosphate biosynthetic process	74	0.0411	*CMPK2*
GO:0010526	Retrotransposon silencing	74	0.0411	*APOBEC3B*
GO:0039692	Single stranded viral RNA replication via double stranded DNA intermediate	74	0.0411	*APOBEC3B*
GO:0032197	Retrotransposition	70	0.0425	*APOBEC3B*
GO:0032069	Regulation of nuclease activity	66	0.0438	*OAS3*

GO term ID	GO Term	Fold Enrichment	Enrichment FDR	Associated Genes Found
GO:0032196	Transposition	66	0.0438	*APOBEC3B*
GO:0034162	Toll-like receptor 9 signaling pathway	62	0.0448	*RSAD2*
GO:0019883	Antigen processing and presentation of endogenous antigen	62	0.0448	*HLA-C*
GO:0002726	Positive regulation of T cell cytokine production	59	0.0448	*RSAD2*
GO:0060339	Negative regulation of type I interferon-mediated signaling pathway	59	0.0448	*OAS3*
GO:0009147	Pyrimidine nucleoside triphosphate metabolic process	59	0.0448	*CMPK2*
GO:0009219	Pyrimidine deoxyribonucleotide metabolic process	59	0.0448	*CMPK2*
GO:0002828	Regulation of type 2 immune response	57	0.0462	*RSAD2*
GO:0001916	Positive regulation of T cell mediated cytotoxicity	52	0.0499	*HLA-C*
GO:0002711	Positive regulation of T cell mediated immunity	48	0.0210	*HLA-C, RSAD2*
GO:0002709	Regulation of T cell mediated immunity	33	0.0300	*HLA-C, RSAD2*
GO:0002456	T cell mediated immunity	25	0.0300	*HLA-C, RSAD2*
GO:0002708	Positive regulation of lymphocyte mediated immunity	25	0.0300	*HLA-C, RSAD2*
GO:0002824	Positive regulation of adaptive immune response based on somatic recombination of immune receptors built from immunoglobulin superfamily domains	24	0.0300	*HLA-C, RSAD2*
GO:0002821	Positive regulation of adaptive immune response	23	0.0300	*HLA-C, RSAD2*
GO:0002705	Positive regulation of leukocyte mediated immunity	21	0.0300	*HLA-C, RSAD2*
GO:0002706	Regulation of lymphocyte mediated immunity	17	0.0300	*HLA-C, RSAD2*
GO:0002753	Cytoplasmic pattern recognition receptor signaling pathway	16	0.0324	*RSAD2, OAS3*
GO:0002822	Regulation of adaptive immune response based on somatic recombination of immune receptors built from immunoglobulin superfamily domains	16	0.0324	*HLA-C, RSAD2*
GO:0019058	Viral life cycle	15	0.0210	*OAS3, APOBEC3B, RSAD2*
GO:0002819	Regulation of adaptive immune response	14	0.0364	*HLA-C, RSAD2*
GO:0002699	Positive regulation of immune effector process	13	0.0375	*HLA-C, RSAD2*
GO:0002703	Regulation of leukocyte mediated immunity	13	0.0375	*HLA-C, RSAD2*
GO:0002221	Pattern recognition receptor signaling pathway	11	0.0411	*RSAD2, OAS3*
GO:0002449	Lymphocyte mediated immunity	11	0.0425	*HLA-C, RSAD2*
GO:0002758	Innate immune response-activating signaling pathway	10	0.0438	*RSAD2, OAS3*
GO:0002460	Adaptive immune response based on somatic recombination of immune receptors built from immunoglobulin superfamily domains	10	0.0448	*HLA-C, RSAD2*
GO:0002218	Activation of innate immune response	9	0.0462	*RSAD2, OAS3*

Supplementary Table ST6: Exploratory ROC performance metrics for baseline (no treatment) *RSAD2* expression in the validation cohort.

Threshold	Sensitivity	95% CI	Specificity	95% CI	Likelihood ratio
< 0.035	0.333	0.152 to 0.583	1.000	0.439 to 1.000	
< 2.430	0.400	0.198 to 0.643	1.000	0.439 to 1.000	
< 4.809	0.467	0.248 to 0.699	1.000	0.439 to 1.000	
< 4.850	0.533	0.301 to 0.752	1.000	0.439 to 1.000	
< 5.194	0.600	0.358 to 0.802	1.000	0.439 to 1.000	
< 6.437	0.667	0.417 to 0.848	1.000	0.439 to 1.000	
< 7.486	0.667	0.417 to 0.848	0.667	0.119 to 0.983	2.000
< 10.66	0.733	0.481 to 0.891	0.667	0.119 to 0.983	2.200
< 15.22	0.800	0.548 to 0.930	0.667	0.119 to 0.983	2.400
< 20.93	0.867	0.621 to 0.976	0.667	0.119 to 0.983	2.600
< 25.53	0.933	0.702 to 0.997	0.667	0.119 to 0.983	2.800
< 33.81	0.933	0.702 to 0.997	0.333	0.017 to 0.882	1.400
< 50.43	1.000	0.796 to 1.000	0.333	0.017 to 0.882	1.500
